# Granger Causality–Based Analysis for Classification of Fibrillation Mechanisms and Localization of Rotational Drivers

**DOI:** 10.1161/CIRCEP.119.008237

**Published:** 2020-02-16

**Authors:** Balvinder S. Handa, Xinyang Li, Kedar K. Aras, Norman A. Qureshi, Ian Mann, Rasheda A. Chowdhury, Zachary I. Whinnett, Nick W.F. Linton, Phang Boon Lim, Prapa Kanagaratnam, Igor R. Efimov, Nicholas S. Peters, Fu Siong Ng

**Affiliations:** 1 National Heart & Lung Institute, Imperial College London, United Kingdom (B.S.H., X.L., N.A.Q., I.M., R.A.C., Z.I.W., N.W.F.L., P.B.L., P.K., N.S.P., F.S.N.).; 2 Department of Biomedical Engineering, George Washington University, Washington, DC (K.K.A., I.R.E.).

**Keywords:** algorithm, atrial fibrillation, catheter ablation, incidence, ventricular fibrillation

## Abstract

The mechanisms sustaining myocardial fibrillation remain disputed, partly due to a lack of mapping tools that can accurately identify the mechanism with low spatial resolution clinical recordings. Granger causality (GC) analysis, an econometric tool for quantifying causal relationships between complex time-series, was developed as a novel fibrillation mapping tool and adapted to low spatial resolution sequentially acquired data.

**Methods:**

Ventricular fibrillation (VF) optical mapping was performed in Langendorff-perfused Sprague-Dawley rat hearts (n=18), where novel algorithms were developed using GC-based analysis to (1) quantify causal dependence of neighboring signals and plot GC vectors, (2) quantify global organization with the causality pairing index, a measure of neighboring causal signal pairs, and (3) localize rotational drivers (RDs) by quantifying the circular interdependence of neighboring signals with the circular interdependence value. GC-based mapping tools were optimized for low spatial resolution from downsampled optical mapping data, validated against high-resolution phase analysis and further tested in previous VF optical mapping recordings of coronary perfused donor heart left ventricular wedge preparations (n=12), and adapted for sequentially acquired intracardiac electrograms during human persistent atrial fibrillation mapping (n=16).

**Results:**

Global VF organization quantified by causality pairing index showed a negative correlation at progressively lower resolutions (50% resolution: *P*=0.006, *R*^2^=0.38, 12.5% resolution, *P*=0.004, *R*^2^=0.41) with a phase analysis derived measure of disorganization, locations occupied by phase singularities. In organized VF with high causality pairing index values, GC vector mapping characterized dominant propagating patterns and localized stable RDs, with the circular interdependence value showing a significant difference in driver versus nondriver regions (0.91±0.05 versus 0.35±0.06, *P*=0.0002). These findings were further confirmed in human VF. In persistent atrial fibrillation, a positive correlation was found between the causality pairing index and presence of stable RDs (*P*=0.0005,*R*^2^=0.56). Fifty percent of patients had RDs, with a low incidence of 0.9±0.3 RDs per patient.

**Conclusions:**

GC-based fibrillation analysis can measure global fibrillation organization, characterize dominant propagating patterns, and map RDs using low spatial resolution sequentially acquired data.

## Abstract 2

Supplemental Digital Content is available in the text.

**What Is Known?**

- Preclinical studies have implicated multiple competing mechanisms for sustaining myocardial fibrillation.
- Clinical translation to guide treatment in patients with atrial fibrillation and ventricular fibrillation survivors remains challenging due to the poor spatial resolution of clinical mapping systems and a lack of suitable analysis tools.

**What this Study Adds?**

- Granger causality analysis, originally an econometric tool for quantifying causal relationships between complex time-series, was developed in rat ventricular fibrillation and validated in human ventricular fibrillation and atrial fibrillation as a novel fibrillation mapping tool.
- Granger causality–based fibrillation analysis can measure global fibrillation organization, characterize dominant propagating patterns, and map rotational drivers using low spatial resolution sequentially acquired data.

The underlying mechanisms sustaining myocardial fibrillation remain unclear, and there is a lack of consensus on a unifying mechanism. The anarchical hypothesis of multiple self-perpetuating wavelets and the opposing hierarchal hypothesis of organized spiral wave reentry, referred to as rotors or rotational drivers (RDs), organized around a nonanatomic unexcited core termed a phase singularity (PS) point, continue to be debated as the mechanism sustaining fibrillation. Although evidence exists from high spatiotemporal resolution optical mapping studies to support these conflicting mechanisms,^1–4^ clinical translation to guide treatment remains challenging due to the poor spatiotemporal resolution of clinical mapping systems and a lack of suitable analysis tools. Thus, outcomes from catheter ablation remain poor, especially in persistent atrial fibrillation (AF), where recurrence remains as high as 40% to 50%.^5^

Multiple prominent investigators have proposed that ablation of sites localizing RDs can terminate AF^6^ and prevent ventricular fibrillation (VF) reoccurrence in survivors of sudden cardiac death.^7^ Others have found no evidence for their existence in fibrillation mapping studies during cardiac surgery with high-resolution electrode arrays^8^ and proposed complex asynchronous endocardial-epicardial disassociation of fibrillatory conduction as a further mechanism.

A challenge in mapping fibrillatory mechanism is its dynamic nature. There is beat-to-beat variability in periodicity and amplitude of signals, and global wavefront propagation is nonuniform and temporally variable. RDs often demonstrate meandering and transient trajectories.^9^ Conventional mapping techniques, such as activation mapping, which require annotation of a reference signal^10^ and stable linear propagation, are poorly suited. To overcome this issue, phase analysis is frequently used in fibrillation mapping instead. Phase analysis assigns a phase value between π and −π to the activation-recovery cycles of a given myocardial area.^11^ Tracking these changes in phase allow for annotation of propagating wavefronts and localization of PSs as areas devoid of a definitive phase. Accurate phase analysis, however, requires both global panoramic mapping and adequate spatial resolution,^12^ which is currently not possible with clinically available tools.

We previously demonstrated that RD localization from phase analysis of intracardiac electrograms acquired by multipolar catheters, including the 64-electrode basket catheters, is inaccurate as it lacks sufficient resolution, requires significant interpolation, stitching of sequentially acquired data and is prone to generation of a high number of false-positive RDs.^13^ Other research groups have also highlighted the limitations of phase analysis with low spatial resolution^14^ and demonstrated an increase in false-positive RD detection rate with increasing interelectrode distance and noise.^15^ In addition, basket catheters can provide incomplete surface coverage and are susceptible to poor contact.^16^ Alternatively, global cardiac mapping noninvasively is possible from the body surface using a multielectrode electrocardiographic imaging vest. Electrocardiographic imaging uses inverse solution algorithms for interpolating intracardiac electrograms. Although there are some limitations with electrocardiographic imaging, such as correlation between surface and contact electrograms^17^ and noise artifact, it does provide higher resolution mapping and has shown potential in a number of early studies where it has been utilized to map AF,^18^ VF,^7^ and ventricular tachycardia^19^ mechanisms.

Here, we propose that Granger causality (GC) analysis, originally an econometric tool designed to determine causal relationships between complex time-series data,^20^ can be repurposed as a novel tool to analyze fibrillation. Given GC analysis depends only on neighboring causal relationships, we postulated that it could overcome the limitation of spatial resolution and sequentially acquired limited coverage data in fibrillation. We generated a range of fibrillation mechanisms in a rat VF model by modulating gap junction coupling and fibrosis, two factors implicated in cardiac remodeling. We hypothesized that GC-based analysis can be used to (1) analyze temporal dependence of fibrillatory signals in neighboring regions and determine the dominant propagating pattern, (2) quantify the global organization and general mechanism of fibrillation, and (3) map stable RDs at low spatial resolution with limited coverage. Initially developed and validated against high-resolution phase analysis in a rat VF model, these novel GC-based analysis tools were further tested in previous VF optical mapping recordings of coronary perfused donor heart left ventricular (LV) wedge preparations and finally adapted to analyze multielectrode catheter recordings of persistent AF patients.

## Methods

The data, analytic methods, and study materials are available from the corresponding author to other researchers for the purposes of reproducing the results or replicating the procedure upon reasonable request. Methods are described briefly here. For full details, please see the Data Supplement.

### Ethical Approval

The animal work was performed in accordance with standards set out in the United Kingdom Animals (Scientific Procedures) Act 1986 and was approved by Imperial College London Ethical Review Board under the project license PEE7C76CD and PCA5EE967. For the clinical component of the study, patients with symptomatic persistent AF presenting for their first ablation to Imperial College Healthcare National Health Service Trust were prospectively enrolled. The study was approved by the Local Research and Ethics Committee, and written informed consent was obtained from all patients. Experiments using human heart tissue were previously approved by the Institutional Review Board (Office of Human Research) at the George Washington University.^21^

### Experimental Protocols

Eighteen Sprague-Dawley rats (250–300 g) were humanely killed, and the hearts were explanted, heparinized, and rapidly perfused ex vivo on a Langendorff apparatus with Krebs-Henseleit solution and stabilized for a 15-minute period before ex vivo optical mapping studies of transmembrane potential. To create a range of VF activity, in group 1, 8 of the hearts were acutely perfused with a gap junction uncoupler, carbenoxolone (0–50 µM), which in our previous experiments produced progressively disorganized VF at increasing doses. In group 2, the other 10 hearts had chronic patchy ventricular fibrosis which had been induced with ischemia-reperfusion cardiac surgery 4 weeks before the experiment (Figure I in the Data Supplement). No drugs were added to the perfusate, and in our previous experiments, patchy fibrosis was found to sustain a more organized form of VF. Programmed electrical stimulation using a burst pacing protocol with the aid of Pinacidil 30 µM was used to induce and sustain VF.

### Optical Mapping

Explanted hearts underwent optical mapping of the epicardial surface of the LV anterior wall after VF induction. The transmembrane voltage was recorded from optical mapping fluorescence data using our custom made complementary metal-oxide semiconductor camera (Cairn Research, Faversham UK) utilizing the potentiometric dye RH237 (25 µL of 1 mg/mL dimethyl sulfoxide; Thermo-Fisher, MA) and excitation-contraction uncoupler blebbistatin (10 µmol/L; Tocris Bio-Sciences, Cambridge, United Kingdom) in 160×128 pixel resolution for a 10-second duration. All our methods for filtering and analyzing optical mapping fluorescence data have been previously described in detail.^22,23^

### Organizational Analysis

The processed optical mapping data were firstly analyzed to quantify the degree of global organization with 2 novel independent methods: causality pairing index (CPI) derived from global GC analysis and frequency dominance index (FDI) derived from the dominant frequency analysis. These indices are described below in detail.

#### GC Analysis

GC analysis is an econometric methodology for quantifying the causal dependence between two or more complex time-series using a linear autoregressive model.^20,24^ GC is a concept based on statistical prediction, whereby GC analysis statistically tests if a given time-series signal A causes time-series signal B, by analyzing information contained in past values of time-series A and determining if the information contained in signal A can predict signal B, beyond predictions from past values of signal B alone. In this work, we developed novel GC-based tools for fibrillation analysis and adapted them for low spatial resolution and limited coverage sequential mapping from optical fluorescence data of transmembrane potentials in a perfused rat VF model. GC analysis was used to measure the strength of the causal relationship between signals in neighboring regions and to quantify whether the fibrillation signal in one region over time could predict signals in another. GC vector mapping (described below) was performed based on quantified strengths of these relationships to determine the dominant propagating patterns.

#### Causality Pairing Index

**Figure 1.**
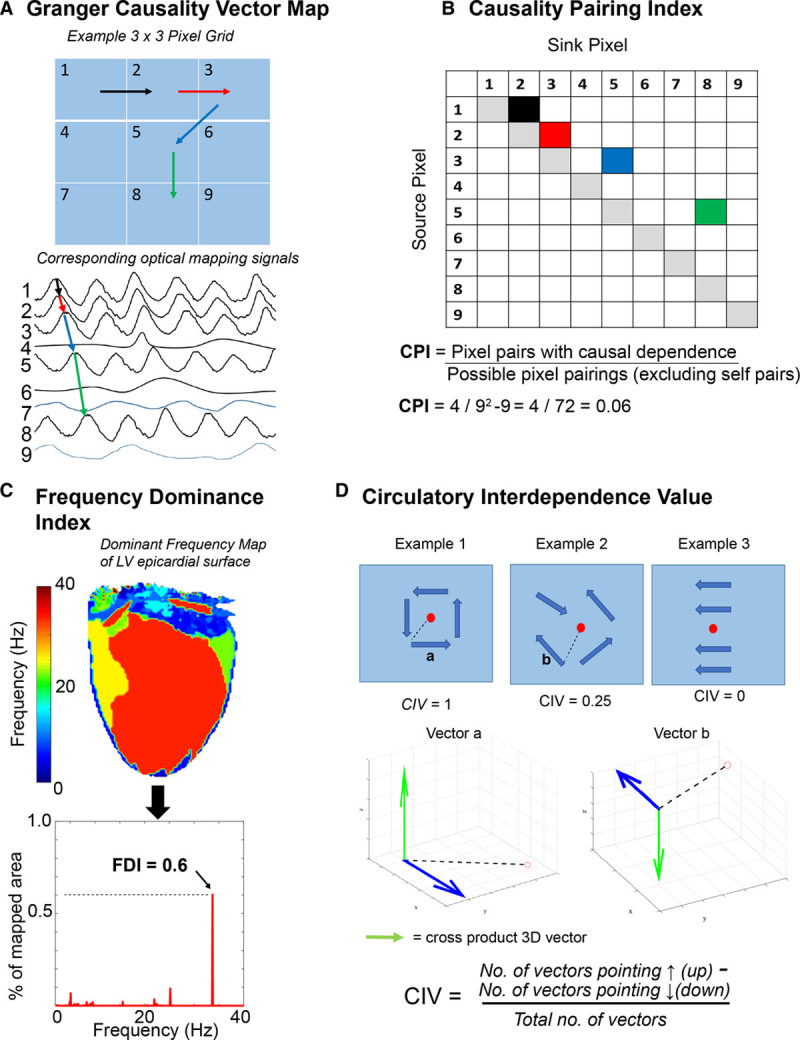
**Novel tools for measuring organization and localizing areas with rotational drivers (RDs).** **A**, An example simplified 3×3 pixel grid with corresponding optical mapping signals below showing Granger causality (GC) vectors between signals with the strongest temporal dependence. **B**, Causality pairing index for data in (**A**) showing 4 pixel pairs with causal temporal dependence of propagation (corresponding to the GC vectors, shaded in color). Causality pairing index (CPI) was defined as the pixel pairs with causal dependence divided by all possible pixel pairings (excluding self-pairs, shaded in gray) in a data set. **C**, An example dominant frequency map showing all the dominant frequencies driving fibrillation in the left ventricular (LV) anterior wall. The histogram below plots these frequencies. The frequency dominance index (FDI) is defined as the proportion of area occupied by the largest organized dominant frequency area in the global fibrillatory spectrum divided by the total area of all regions with a defined dominant frequency (black arrow). **D**, Circulatory interdependence value (CIV) for 3 examples. For each GC vector (dark blue), a cross-product virtual 3-dimensional (3D) vector was generated (green arrow) relative to vector to the center (dashed black line). The resulting virtual 3D vector was binarized as pointing up or down as shown in example for vector a and vector b. CIV was calculated on a scale of 0 to 1 by subtracting the number of vectors pointing down from no of vectors pointing up divided by total number of vectors. The 3 examples above demonstrate expected values for example 1: stable RD, example 2: random propagation, example 3: linearly propagating wavefront. With this method areas of rotational activity will give a CIV value closer to 1.

CPI calculates global organization of fibrillation from GC analysis, as described above. In this study, the temporal dependence structure between signals from different pixels was calculated by fitting a vector autoregression model to a multivariate signal. Thereafter, the CPI was measured by quantifying the percentage of possible pixel pairings between which there are propagational effects on a normalized scale of 0 to 1, where 0 is defined as no possible pairing having causal dependency and 1 where all possible pairings have causal dependency (Figure 1A and 1B). The more pixel pairings that have a propagational effect between them above a specified threshold, the greater the level of global organization in fibrillation.

#### Frequency Dominance Index

The FDI calculates the total level of global organization by analyzing all the dominant frequencies from all the signals within a fibrillating ventricle. The FDI is defined as the proportion of area occupied by the largest organized dominant frequency area in the global fibrillatory spectrum relative to the total area of all regions with a defined dominant frequency (Figure 1C). The methodology for calculating dominant frequency has been previously described in detail.^25^

### Downsampling Spatial Resolution

After quantifying the level of global organization in the VF data with CPI and FDI, we tested whether these indices were reliable and adaptable to lower spatial resolutions. CPI and FDI were benchmarked at decreasing spatial resolution (with data downsampling) against 2 measures of global fibrillatory organization, locations occupied by phase singularities (l_ps_) and number of rotations (n_r_)/locations occupied by a rotational driver (l_r_), generated from full spatial resolution phase processed data.

### Phase Mapping

Rat VF phase processed analysis from full spatial resolution optical mapping was used for fully characterizing the underlying mechanism of fibrillation and for benchmarking our novel GC-based tools. Our methods for phase analysis and tracking of RDs have previously been described in detail.^25,26^ A phase map of VF at each sampled time point was constructed and PS tagged using our algorithm. The edge of each wavefront was tracked in a 9×9 pixel window and maximum number of rotations (max [n_r_]) calculated. A minimum 2-rotation filter was used to threshold and define a significant RD and to construct RD heat maps from full spatial resolution data for validating our methodology for fibrillation analysis.

#### Phase Characterization of Organization and Stability

From phase processed fibrillatory data, rotational activity was quantified by our metrics of organization and stability (n_r_/l_r_ and l_ps_) and compared with CPI, FDI, and a more widely used analysis feature in fibrillation literature, Shannon entropy (Sh_en_).^27^ PS with <2 rotations were labeled nonsignificant PSs, and the number of locations (pixels) they occupied (l_ps_) acted as a measure of global disorganization, spatial meander, and instability, whereby a large number of short-lived meandering PSs would generate the highest value by this metric. PSs with ≥2 rotations were labeled significant RDs, the number of rotations they exhibited (n_r_) and the number of locations (pixels) they occupied (l_r_) over a fibrillatory recording were tracked; thus n_r_ divided by l_r_ acted as a measure of stability and global organization, whereby RDs with high number of rotations localizing to a small area would generate the highest values of this metric. These objective measures of fibrillation organization (n_r_/l_r_ and l_ps_) calculated from high-resolution phase analysis were correlated with our novel low-resolution measures of fibrillation organization (CPI and FDI). In addition, we correlated a conventional fibrillation analysis tool, Sh_en_,^25,27^ with CPI and FDI.

### GC Vector Maps

GC vector mapping was tested as a low spatial resolution tool adapted specifically for limited spatial coverage to determine dominant propagation and localize RDs independent of phase analysis. In this work, GC vector maps were generated from an 8×8 data grid at 25% of the full spatial resolution optical fluorescence data, whereby 3 pixels were discarded between each data point. Within this 8×8 data grid, an algorithm determined firstly if there were any signals with temporal causal dependence, thereafter it quantified the strength of the temporal causal dependence between these signals. A vector was then plotted only between the source signal and the signal where it exerted the greatest causal or propagational effect above a specified threshold (Figure 1A). GC vector maps were benchmarked against full-resolution phase processed data to determine ability to characterize fibrillation mechanism.

A circular interdependence value (CIV) is proposed to quantify the circular interdependence of signals in the local 8×8 GC data grid and identify the location of RDs. For each GC vector, we calculated the cross-product relative to the center of the grid using the right-hand rule (Figure 1D). The resultant cross-product vector was binarized as either pointing up or down in a virtual 3-dimensional (3D) space. Using this principle, 3 possibilities exist: (1) an organized continuous circular one-directional rotation of GC vectors over time would generate all virtual cross-product 3D vectors in the same direction (either up or down; 2) GC vectors with a disorganized arrangement would generate cross-product 3D vectors in both directions (up and down) dependent on degree of disorganization; (3) GC vector all in one direction, for instance, a propagating linear wavefront, would also generate cross-product 3D vectors in both direction. Thus by applying the equation below to these 3D vectors outputs the CIV can be calculated.


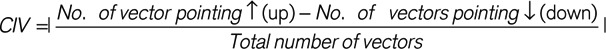


CIV is quantified on a normalized scale of 0 to 1, whereby a stable RD present throughout the recording would generate a value of 1, and disorganized or propagating wavefronts in one direction will generate a value closer to 0.

### Ex Vivo Human VF Mapping

Our GC-based analysis tools were tested on human VF optical mapping data that was previously acquired as part of a separate study by Aras et al,^21^ and the methodology was reported in detail. Briefly, we tested our GC-based analysis tools on 33 VF recordings from 12 representative deidentified human donor hearts. These recordings were 4-second in duration and taken from coronary perfused LV wedge preparations that had VF induced with 25 µM pinacidil pretreatment. The mean LV wedge dimensions were 7 cm×3.5 cm×1.8 cm (height×width×thickness).

### Clinical In Vivo AF Mapping

In 16 patients presenting with symptomatic persistent AF for a first ablation procedure, electrograms were acquired using a 20-pole double-loop catheter (InquiryTM AFocusII; St Jude Medical, MN) with 4 mm electrode spacing. The term kernel defined an area or location of atrial myocardium mapped that is subtended by the AFocusII mapping catheter. The data was imported from Ensite Velocity into MATLAB R2018 (MathWorks, MA) using a custom made script. Twenty seconds of bipolar electrogram data were processed with bandpass (40–250 Hz) and low-pass filtering (with a cut off <25 Hz) and followed by signal rectification. The entire recording was used for organizational analysis with CPI. GC vectors were plotted for each kernel and the CIV calculate to localize RDs. CIV threshold for localizing RDs was established as 0.61 by plotting rat VF data on a receiver operating characteristic curve. To allow for meandering of RDs temporally, 8-second windows with overlapping window-shifts of 1-second was applied to segment the electrogram data.

### Statistical Analysis

All statistical analysis was performed using a statistics software package (Prism version 5; Graphpad Software, CA) or MATLAB. After normality testing, Student *t* tests were used to compare means between 2 groups. For each optical mapping recording, objective measures derived from optical mapping analysis were calculated, together with FDI, CPI, and Sh_en_. Linear regression models were fitted to FDI, CPI, or Sh_en_ as explanatory variables and l_ps_ or n_r_/l_r_ as response variables, and *F* test was applied for the linear models. R-squared measures were applied to test the strength of the relationship between the model and the dependent variable. *P*<0.05 was regarded as significant. Results are expressed as mean±SEM.

## Results

### Fibrillation Organization Quantified by the FDI and CPI

**Figure 2.**
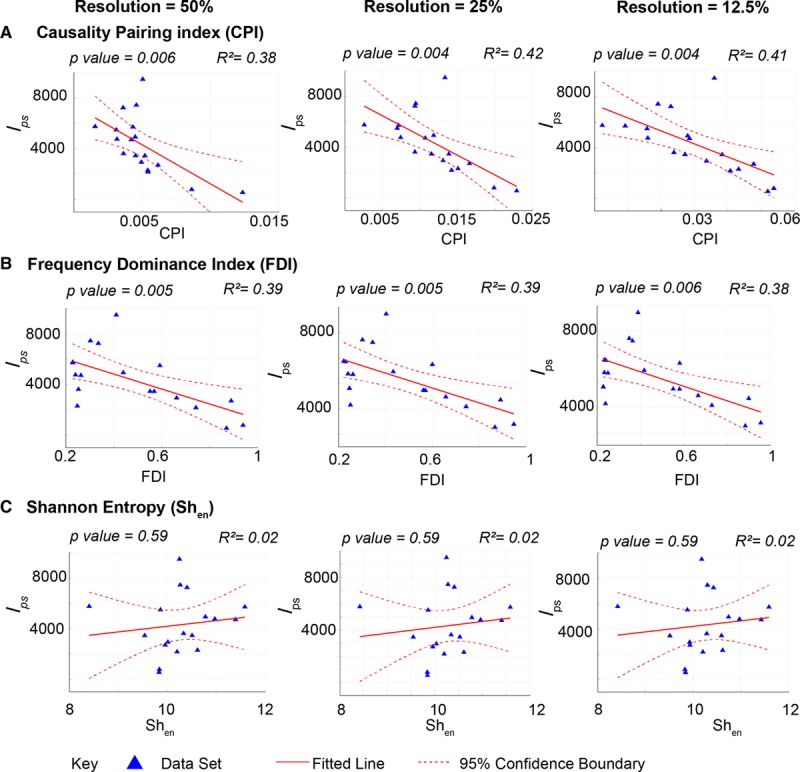
**The causality pairing index (CPI) and frequency dominance index (FDI) can characterize the global organization of fibrillation at low spatial resolution.** Graphs showing negative correlation between a measure of disorganization and instability, the number of locations occupied by nonsignificant short-lived phase singularities (PSs; locations occupied by PSs [l_ps_]) and CPI (**A**) and l_ps_ and FDI (**B**), and no correlation between l_ps_ and Shannon entropy (Sh_en_; **C**), at decreasing resolutions of 50% (**left**), 25% (**middle**), and 12.5% (**right**) of full spatial resolution from optical mapping of rat ventricular fibrillation. Nonsignificant PSs were defined as PSs with <2 rotations, and rotational drivers were defined as >2 rotations. Linear regression analysis, *F* test, coefficients of determination–*R*^2^ and *P* values are indicated, n=18.

Rat VF optical fluorescence data were recorded in hearts with underlying chronic fibrosis, or acute GJ uncoupling with carbenoxolone. In previous experiments, with high-resolution phase processed data, a spectrum of fibrillatory mechanisms was found in these hearts, ranging from fibrillation driven by organized RDs to completely chaotic activity. At decreasing spatial resolutions, l_ps_, a measure of global disorganization correlated negatively with both CPI (50% resolution: F[1,16]=9.9, *P*=0.006, *R*^2^=0.38, 25% resolution: F[1,16]=11.7, *P*=0.004, *R*^2^=0.42 and 12.5% resolution F[1,16]=11.0, *P*=0.004, *R*^2^=0.41) and FDI (50% resolution: F[1,16]=10.4, *P*=0.005, *R*^2^=0.39, 25% resolution: F[1,16]=10.4, *P*=0.005, *R*^2^=0.39 and 12.5% resolution F[1,16]=9.8, *P*=0.006, *R*^2^=0.38; Figure 2A and 2B). Disorganized fibrillation with a high number of meandering nonsignificant PSs had low FDI and CPI values. Conversely, n_r_/l_r_, a measure of global fibrillatory organization that tracks presence of spatiotemporally stable RD from full-resolution phase processed data, correlated positively with CPI and FDI at decreasing resolution (Figure IIA and IIB in the Data Supplement). Sh_en_ values, more conventionally utilized in fibrillation analysis, showed no statistically significant correlation with l_ps_ or n_r_/l_r_ (Figure 2C, Figure IIC in the Data Supplement).

**Figure 3.**
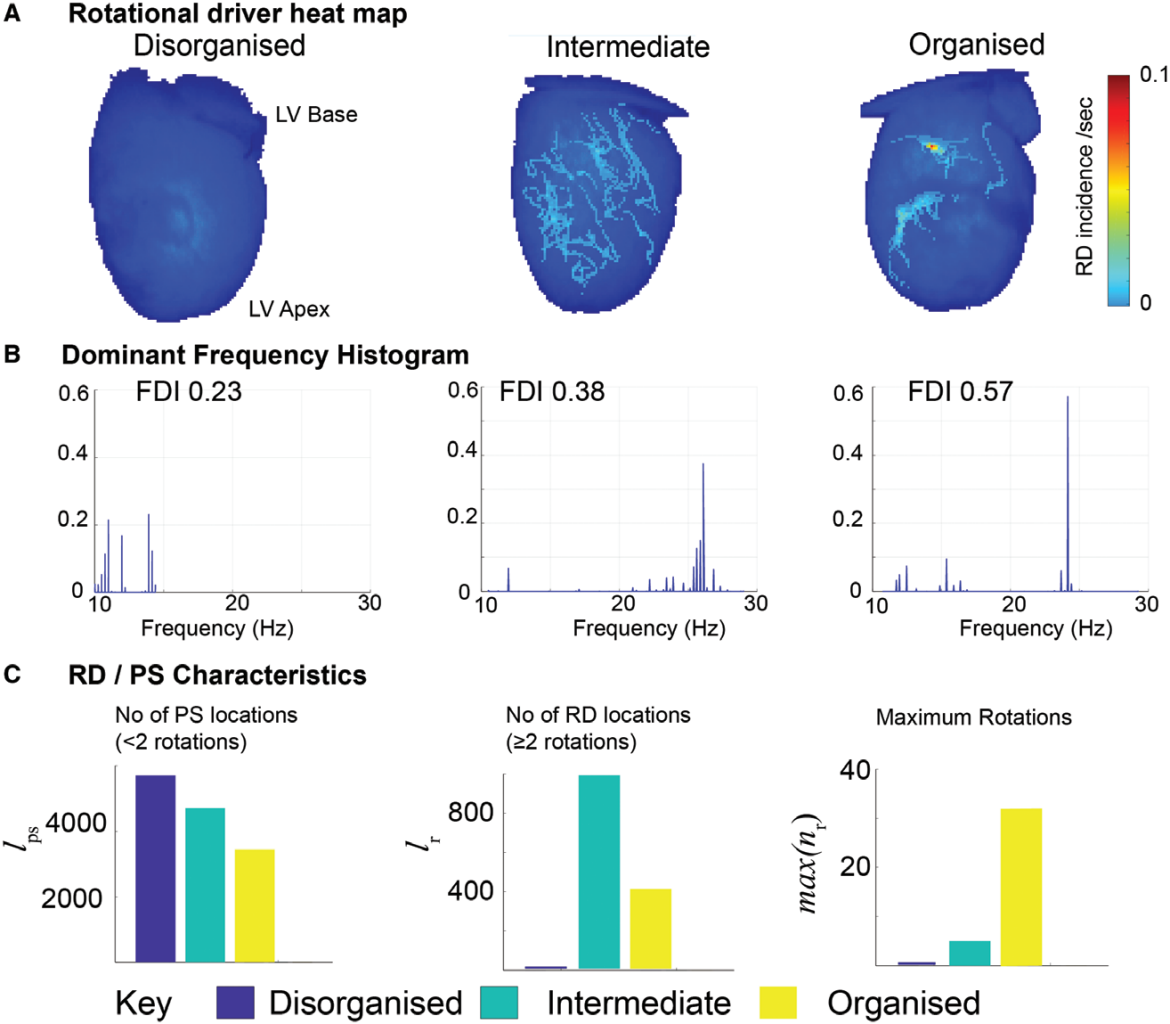
**Quantifying global organization in fibrillation infers the likely underlying mechanism.** Representative data sets of rat ventricular fibrillation selected from organizational analysis categorization of fibrillation as low (**left**), intermediate (**middle**), and organized (**right**) from Figure 2. **A**, Rotational driver (RD) heat map showing incidence of significant RDs (≥2 rotations). **B**, The respective global dominant frequency histogram with frequency dominance index (FDI) value and (**C**) graphs showing characterization of RD for each data set, l_ps_–number of locations/pixels occupied by nonsignificant phase singularity [PS] with <2rotation, l_r_–number of locations occupied by significant RDs with ≥2 rotations, max [n_r_]–maximum rotations for a single significant RDs. LV indicates left ventricular.

After determining the applicability of our novel organizational indices to low-resolution data, we selected representative hearts along the organizational spectrum, to delineate whether the fibrillatory mechanism characterized by full-resolution phase analysis correlated with the level of global organization as determined with CPI and FDI with low spatial resolution data. Hearts with the highest FDI had the most spatiotemporally stable RDs (Figure 3A and 3B), with high numbers of rotations and much fewer short-lived PSs in comparison to hearts with a lower FDI (Figure 3C). The RD heat map showed well-localized discrete areas harboring the organized RD in hearts with a high FDI, whereas hearts with a low to intermediate FDI values did not show such areas (Figure 3A).

### GC Mapping to Localize Driver Regions

**Figure 4.**
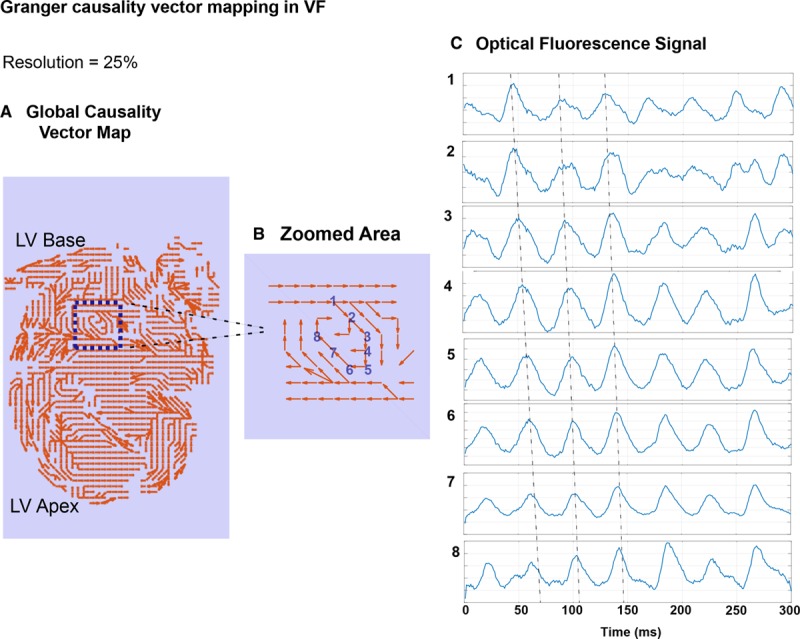
**Granger causality (GC) mapping can be used for analyzing fibrillation data.** An example GC vector map of an organized rat ventricular fibrillation (VF) heart showing neighboring regions with causal interdependence (**A**) and zoomed localization of a driver region showing a signature continuous circular interdependence of neighboring GC vectors (**B**), with correlating optical mapping signals from the driver region showing repetitive sequential activation (**C**). Data analyzed at 25% of full spatial resolution, correlating rotational driver heat map in Figure 5—heart C. LV indicates left ventricular.

After establishing that high global organization in fibrillation, as measured by CPI and FDI, correlated positively with the existence of localized, stable RDs, we tested whether GC vector mapping could identify causally dependent neighboring regions to localize areas harboring RDs at low spatial resolutions independent of phase analysis. In a representative heart with high global fibrillatory organization, optical fluorescence data was downsampled to 25% of full spatial resolution for GC vector mapping. GC vector mapping identified a number of regions with causal dependence and localized an area harboring a spatiotemporally stable RD with a circular interdependence of GC vectors, as shown in Figure 4A and 4B. Optical fluorescence of the transmembrane potentials along this region showed repetitive sequential activation over time (Figure 4C). At 12.5% of the full spatial resolution, RD regions could no longer be localized accurately with GC mapping (results not enclosed).

**Figure 5.**
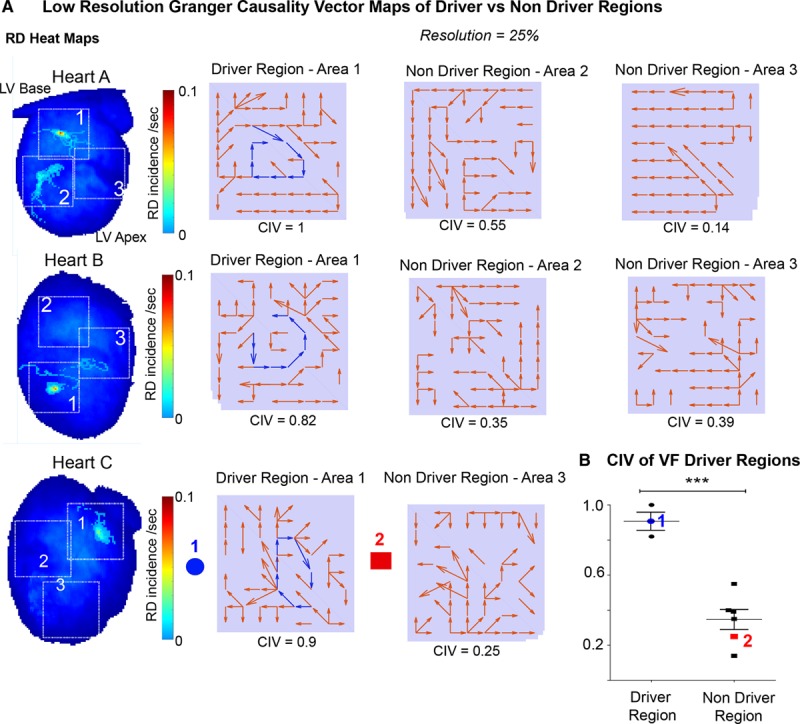
**Granger causality (GC) vector mapping can reliably localize and differentiate between areas harboring rotational drivers (RDs) and areas without RDs in ventricular fibrillation (VF).** **A**, RD heat maps constructed from 3 organized rat VF data sets; heart A, B, and C (**left**) and the correlating limited coverage GC vector maps showing driver regions with continuous circular interdependence of GC vectors with 1-directional flow of GC vectors (blue) and nondriver regions showing no circular interdependence of GC vectors. Correlating circular interdependence value (CIV) values between 0 (minimum) and maximum (1) for each respective region listed below. Data analyzed at 25% of full spatial resolution. **B**, A graph showing CIV value of driver regions vs nondriver regions with sample GC maps (**left**, heart **C**). *t* test, n=3, *P*=0.0002. LV indicates left ventricular.

We further validated RD localization with GC vector mapping performed at 25% spatial resolution against full spatial resolution phase analysis in a further 3 hearts classified as organized from high FDI and CPI measures. Figure 5 shows GC vector mapping from these hearts, where regions with circular interdependence of signals had high CIV values (1, 0.82, 0.9), and these regions highly correlated with regions localizing stable RDs on full-resolution phase analysis. However, in nondriver regions where CIV values were low, GC vectors had a random or noncircular distribution, such as heart B area 2 (CIV=0.35). Areas with meandering RDs with less stability showed intermediate organization and an intermediate CIV value, such as heart A area 2 (CIV=0.55, Figure 5A). In regions harboring stable RDs, the CIV was 2.6-fold higher than regions without RD (0.91±0.05 versus 0.35±0.06, *P*=0.0002, n=3, Figure 5B).

### GC-Based Analysis of Human VF

**Figure 6.**
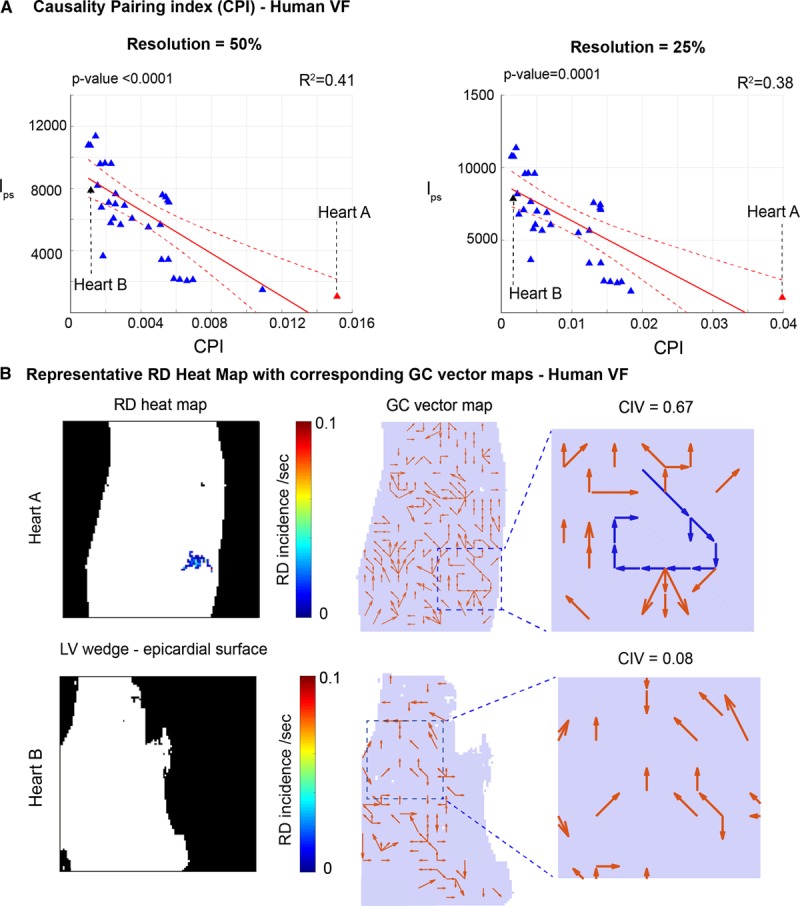
**Granger causality (GC)–based analysis of human ventricular fibrillation (VF) quantifies global fibrillatory organization and maps underlying mechanism.** **A**, Graphs showing negative correlation between locations occupied by phase singularities and causality pairing index (CPI) at decreasing spatial resolution. **B**, Full spatial resolution rotational driver (RD) heat maps in VF of left ventricular (LV) wedge epicardial recordings with corresponding GC vector maps at 25% spatial resolution from 2 representative hearts above (heart A and Heart B). Linear regression analysis, *F* test, coefficients of determination–*R*^2^ and *P* values are indicated, data from 33 VF recordings, n=12. CIV indicates circular interdependence value.

We further tested and validated our low spatial resolution adapted GC-based tools in optical mapping of human VF in LV wedge preparations to test applicability to a larger spatial scale and benchmarked these tools against full-resolution phase analysis. As with rat VF, a spectrum of VF mechanisms were found, ranging from fibrillation driven by organized RDs to completely chaotic activity. At decreasing spatial resolutions, l_ps_ correlated negatively with CPI as before; 50% spatial resolution: F(1,10)=24.4, *P*<0.0001, *R*^2^=0.42 and 25% spatial resolution: F(1,10)=21.3, *P*=0.0001, *R*^2^=0.38 (Figure 6A). As before, the optical fluorescence data was downsampled to 25% of full spatial resolution for GC vector mapping. Figure 6B shows 2 representative hearts with low and high global organization, respectively. In a representative heart with a high global fibrillatory organization, as quantified by CPI, GC vector mapping identified a regions with high CIV, harboring a stable RD and this correlated with the same region identified by phase analysis on the RD heatmap. Similarly, in a representative heart with low global fibrillatory organization, as quantified by CPI, GC vector mapping showed random vector distribution with low CIV values, and this correlated with multiple wavelet–driven fibrillation on phase analysis with no stable RDs.

### GC Vector Mapping of Intracardiac Electrograms From AF Mapping

**Figure 7.**
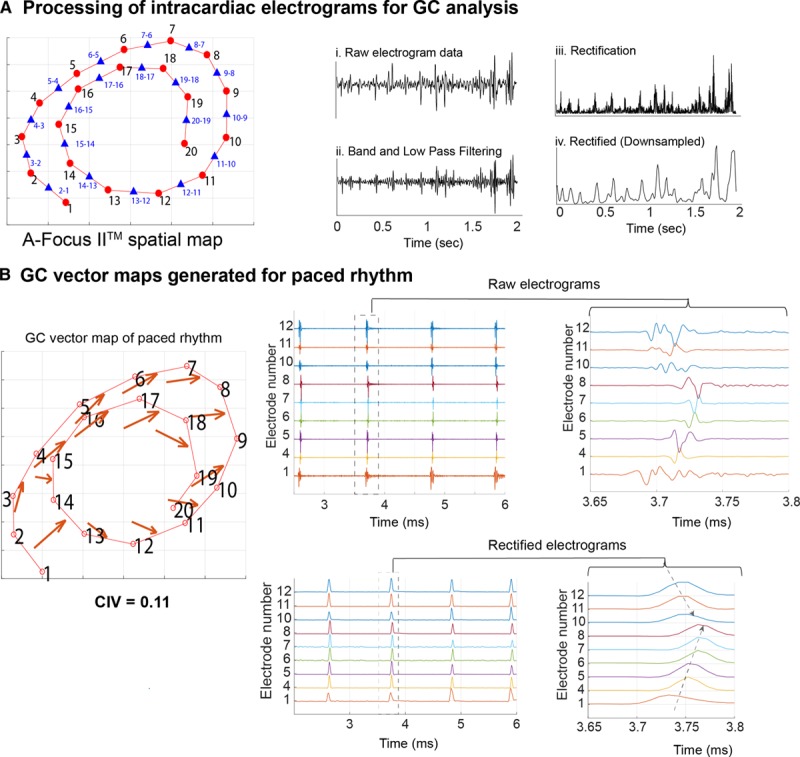
**Granger causality (GC) vector maps generated from intracardiac electrograms acquired with multipolar catheters.** **A**, AFocusII mapping catheter 3-dimensional electrode (red dot) spatial configuration with corresponding bipoles (blue triangle) within the atrium (**left**). Electrograms processing for GC analysis (**right**)—(1) Sample raw bipolar electrograms, (2) 40–250 Hz bandpass filtering and low-pass filtering of signals <25 Hz, (3) signal rectification, (4) downsampling. **B**, Representative GC vector map for a paced rhythm mapped by the catheter (**left**) and correlating raw and rectified electrograms (**right**). CIV indicates circular interdependence value.

After developing and validating these novel GC-based tools for use with low-resolution data from optical mapping of transmembrane voltage, we adapted our methodology to process intracardiac electrograms in human persistent AF acquired sequentially using a 20-pole AFocusII mapping catheter. We measured global fibrillatory organization and localized RDs in persistent AF with our novel indices. Processing electrograms for GC analysis requires different considerations for signal processing than optical fluorescence data. First, a 3D spatial map was constructed from spatial correlates of the electrodes and corresponding bipoles. Each signal underwent high and low bandpass filtering before been rectified and downsampled. Causal dependence between bipoles was established from rectified downsampled data (Figure 7A). Figure 7B demonstrates validation of this methodology with paced data with a wavefront emerging from near electrode 1, 2 and propagating towards 7, 8. If causal dependence between bipoles was present, it was shown with GC vectors (red arrows). The CIV of 0.11 for this representative paced data as expected is low.

### GC Vector Mapping Quantifies Global AF Organization and Identifies Areas Harboring RDs

**Figure 8.**
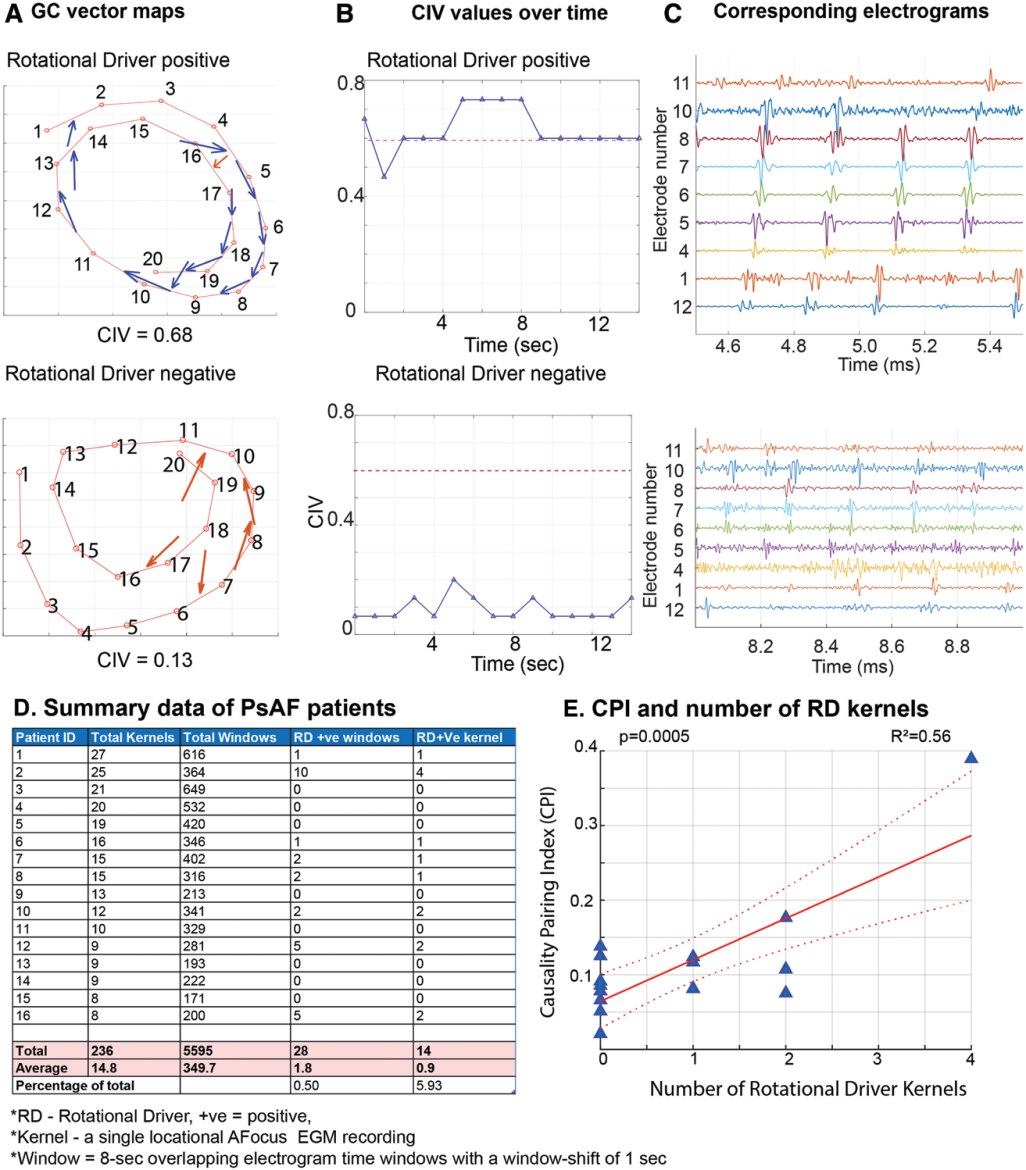
**Granger causality (GC) vector maps can localize rotational drivers (RDs) from intracardiac electrograms acquired with a multipolar catheter.** Representative GC vector maps for an RD-positive site with high circular interdependence value (CIV) value (**top**) and RD negative site with low CIV value (**bottom**), corresponding (**B**) CIV values over time and (**C**) electrograms. *Dashed line=cutoff for a RD-positive site. **D**, Atrial fibrillation (AF) mapping data from 16 persistent AF patients showing the number of kernels and windows with RDs. **E**, Graph showing the positive correlation between causality pairing index (CPI) and number of RDs. Linear regression analysis, *F* test, coefficients of determination–*R*^2^ and *P* value is indicated, n=16. Kernal denotes a single locational AFocus electrogram recording; Window denotes 8-s overlapping electrogram time windows with a window-shift of 1 s. PsAF indicates persistent AF.

The electrogram recordings during AF mapping were processed and causality maps plotted from 20 seconds of recording. The threshold value for an RD-positive site was determined from a receiver operating characteristic curve (Figure III in the Data Supplement). Figure 8A demonstrates a recording that was positive for an RD, where a circular interdependence of GC vectors can be seen between bipoles. The corresponding CIV value shows a small degree of fluctuation over time, suggesting low meander and remains above the threshold value for an RD. The electrograms demonstrate sequential activation between electrodes with causal dependence and rotational configuration over time (Figure 8B and 8C). On the contrary, in sites testing negative for RDs, (Figure 8A–8C) few electrodes demonstrate causal dependence, and the vectors are randomly arranged. The CIV remains low and below the threshold for an RD throughout the recording period, and the corresponding electrograms demonstrate chaotic activity with no discernible underlying patterns of activation.

To take into account the transient and meandering nature of RDs, the electrogram recordings were segmented in 8-second overlapping windows in addition to analysis of the entire 20-second segment to detect RD-positive sites and time windows The term kernel describes a recording set from an area subtended by a multipolar catheter. In the 16 patients with persistent AF, on average, 14.8±1.5 kernels and 349.7±36.6 overlapping 8-second windows of recording were taken from each patient. Using GC vector based identification of RDs, 50% of patients were found to have one or more RDs. Overall, persistent AF was found to be a largely disorganized rhythm, with a low incidence of stable RDs, with only 0.9±0.3 kernels and 1.8±0.7 8-second windows showing an RD per patient (Figure 8D). As seen in the ex vivo experiments above, the data demonstrate a spectrum of organization underlying persistent AF as quantified by the CPI analysis. We found a positive correlation between the CPI values and presence of RDs (F[1,14]=20.3, *P*=0.0005, *R*^2^ 0.56, Figure 8E). In patients with low CPI fibrillation, the underlying mechanism was chaotic activity with no RDs, whereas in patients with higher CPI values, there was a higher number of RDs.

## Discussion

In this study, we developed novel GC-based tools for fibrillation analysis adapted for use with low spatial resolution data acquired with limited coverage sequential mapping, independent of conventionally used phase analysis. We demonstrated that neighboring regions in fibrillation demonstrate causal dependence and that quantifying these causal relationships can determine global fibrillatory organization using our novel indices such as CPI. We showed that high global fibrillatory organization, as measured with our novel indices CPI and FDI, positively correlated with existence of stable RDs. By interrogating causal relationships between neighboring regions with GC vectors, we were able to show a continuous circular interdependence of GC vectors as the hallmark of areas harboring stable RDs. We lastly developed a quantitative tool, the CIV, which could differentiate regions with high density of stable RDs activity, transient and meandering RDs, and nondriver regions. These GC-based tools were developed for nonpanoramic, low-resolution, and sequential limited coverage mapping, validated against high-resolution phase analysis of rat VF and human VF optical mapping data and then adapted for use with electrograms from patients with persistent AF.

Although certain triggers, such as ectopic firing from pulmonary veins in AF^28^ and His-Purkinje system in VF,^29^ are well established, a key challenge in treating fibrillation is the difficulty in accurately determining the underlying fibrillatory mechanism and identifying putative drivers with clinical mapping systems that have substantially lower spatial resolutions than optical mapping and multielectrode array mapping used in preclinical research. This has led to conflicting data and multiple mechanisms being implicated. The initial mathematical model of multiple self-perpetuating wavelets with no clear drivers, proposed by Moe et al,^30^ has been supported by a number of preclinical and clinical studies.^8,31,32^ More recently, this hypothesis has evolved with evidence supporting more complex 3D mechanisms of asynchronous activation, connections, and wave breaks between the endocardium and epicardium in human AF mapped with high-density electrode arrays during cardiac surgery.^8,33,34^ However, some investigators continue to propose that there are regions of high spatiotemporal organization driving fibrillation and cite acute termination of AF through targeted ablation of sites harboring RDs as strong evidence for this hypothesis.^35,36^

The clinically available fibrillation analysis tools, most widely available of which is phase analysis, considered to be the “gold-standard,” have significant limitations. In AF simulation studies, we previously demonstrated that the spatial resolution of multiple commercially available clinical catheters including the AFocusII used in this study is prone to generating a large number of false-positive RD detections from phase processed data and is ineffective at locating RDs with meandering trajectories. In perfused heart AF mapping studies, phantom RDs often appear as spatial resolution is reduced.^37^ Phase mapping additionally requires careful consideration of a number parametric inputs; such as rotational thresholds for labeling RDs and average fibrillation cycle lengths for accurate analysis.^38^

GC-based analysis negates the issues generated by low spatial resolution phase analysis and dynamic nature of fibrillation by considering only causal interdependence of neighboring areas over time rather than attempting to construct panoramic videos of wavefront dynamics within a chamber from interpolated, temporally stitched and sequentially acquired data to determine mechanism. Furthermore, by determining only causal relationships in neighboring regions over long periods of mapping in fibrillation, where activation patterns are dynamic and vary beat to beat, GC mapping also addresses a key limitation of activation mapping in fibrillation, which is annotation of relative activation times from complex electrograms. GC analysis was initially developed as an econometric tool, it has been used to characterize the causal connectivity between different brain regions based on neuroimaging data^39,40^ and is widely used in climate science to establish causal relationships between 2 parameters.^41^

By quantifying the circular interdependence of GC vectors that characterizes an RD with CIV through measurements of the directionality of cross-product vectors relative to the center, we provide a nonsubjective measure for localizing RDs that does not rely on user interpretation. Windowing CIV in time segments can quantify the temporal stability of RDs, accounting for their transient and meandering nature, and determine their overall contribution to the fibrillation mechanism.

We found a direct correlation between global fibrillatory organization and the underlying mechanism in both rat VF and human VF and, therefore, classifying this organization with FDI or CPI in low spatial resolution without detailed mapping may help classify the predominant mechanism and guide treatment strategy. For instance, the operator may choose to pursue detailed mapping and RD ablation in patients with high FDI and CPI values only. Some clinical studies support the existence of a spectrum of organization and mechanisms in fibrillation. For instance, in VF mapping studies of patient undergoing cardiac surgery stable RDs, meandering RDs and multiple wavelets were all found as predominant mechanisms in different patients.^42^ Similarly in AF, noninvasive mapping with electrocardiographic imaging has shown coexistence of a number of mechanisms and varying fibrillation complexity.^18^ The global fibrillatory organization and incidence of RDs were low in many of our persistent AF mapped patients. This suggests that mechanism guided ablation may only be suitable in a select number of persistent AF patients.

Although in our study we found evidence for existence of RDs with continuous circular organization of GC vectors in persistent AF, it is important to point out that a biatrial mapping study of persistent AF patients using high-resolution 512-electrode grid during open-heart surgery failed to show existence of stable RDs.^43^ However, in keeping with our findings, another similar study conducted during open-heart surgery with a 128-electrode grid found a similar spectrum of mechanisms, ranging from disorganized activity through to transient RDs.^44^ Both these studies only involved activation sequence analysis rather than a more sophisticated methodology specifically adapted for localizing RDs. It is probable that RDs may not have been localized due to a lack of robust tools, such as the ones proposed in this work.

Other investigators have taken a similar approach to ours in characterizing mechanisms by considering fibrillatory conduction as propagation within a communication network where neighboring regions exert influence over each other over time. Quantifying and mapping this functional connectivity using mutual information analysis^45^ has, therefore, been explored in AF, although in a differing context, whereby greater connectivity or organization was found in patients with successful ablations. A parallel probabilistic analysis technique looking at proportion of time neighboring signals precede each other called Stochastic Trajectory of Ranked Signals has been used to target driver AF drivers with ablation and produced some promising initial results.^46^ GC-based analysis of AF mapping data has been utilized to identify dominant excitation patterns^47^; however, ours is the only study that uses it to quantify organization of fibrillation and localize RDs and was validated against high-resolution phase analysis. Other techniques exist, specifically divergence and curl mapping, for quantitatively identifying focal and RDs. Although, the methodology was developed for analyzing conduction velocity vectors from activation mapping, it could also be applied to GC vector maps for analyzing fibrillation data.^48^

This study has a few important limitations. GC vector mapping was used to analyze 2-dimensional fibrillatory data and may not reflect the transmural propagation in fibrillation. Electrograms show sharp deflection in fibrillation, rather than a sinusoidal waveforms seen from optical fluorescence. GC-based analysis is more dependent on determining causal relationships over time than timing of local activation, further work is needed to evaluate whether GC vector maps from electrograms and optical fluorescence are comparable. GC-based analysis is used to determine both global fibrillatory organization with CPI and to localize RDs with GC vector maps; thus, these methods are not mutually exclusive and may influence each other. One of the limitations of human VF mapping in this study was that the analysis was not performed in a whole intact ex vivo perfused heart. Nevertheless, the volume of an LV wedge preparation was clearly sufficient to sustain VF, and the volume of myocardium in the wedge preparation greater than the wavelength volume of the fibrillation^21^ and, therefore, a suitable preparation to study fibrillatory dynamics and to validate of the GC-based analysis tools in large hearts.

## Conclusions

In summary, we present novel methodologies based on GC analysis for measuring global fibrillatory organization and mapping RDs. The techniques presented here are optimized for sequential mapping with limited spatial resolution and coverage and were developed and validated against high-resolution phase processed optical mapping data. They were further tested in human VF and then adapted for use with intracardiac electrograms. GC-based fibrillation analysis holds potential for identifying patients with globally organized fibrillation, mapping fibrillation mechanisms, and for guiding ablation therapy within the spatiotemporal constraints of current clinical mapping technology.

## Sources of Funding

This work was supported by the British Heart Foundation (Grants Nos. RG/16/3/32175 and PG/16/17/32069). Dr Ng was also supported by the National Institute for Health Research (NIHR) Imperial Biomedical Research Centre, and an NIHR Clinical Lectureship (CL-2011-21-001). Dr Aras and Prof Efimov acknowledge the support of the Leducq Foundation (project RHYTHM).

## Disclosures

Drs Handa, Li, and Ng and Prof Peters are applicants on a patent to Granger Causality Fibrillation Mapping (UK Patent Application No. 1903259.8). The other authors report no conflicts.

## Supplementary Material


