# Supplementary material for: Granger Causality–Based Analysis for Classification of Fibrillation Mechanisms and Localization of Rotational Drivers
Source: Circ Arrhythm Electrophysiol. 2020 Feb 16;13(3):e008237. doi: 10.1161/CIRCEP.119.008237 (PMC7069398; doi:10.1161/CIRCEP.119.008237)
Supplement: Supplementary file 1 [file hae-13-e008237-s001.pdf]

SUPPLEMENTAL MATERIAL

Supplementary Methods

Ethical Approval

The animal work was performed in accordance with standards set out in the United Kingdom Animals (Scientific Procedures) Act 1986 and was approved by Imperial College London Ethical Review Board under the project license PEE7C76CD and PCA5EE967. For the clinical component of the study, patients with symptomatic persistent AF presenting for their first ablation to Imperial College Healthcare NHS Trust were prospectively enrolled. The study was approved by the Local Research and Ethics Committee for Imperial College Healthcare NHS Trust and written informed consent were obtained from all patients. Experiments using human heart tissue were previously approved by the Institutional Review Board (Office of Human Research) at the George Washington University [1].

Experimental Protocols

Eighteen Sprague-Dawley rats (250-300g, Charles River, Harlow, UK) were humanely killed and the hearts were explanted and Langendorff perfused for ex-vivo optical mapping studies of the transmembrane potential. VF was induced with programmed electrical stimulation (PES). To generate different levels of fibrillation organisation, 8 normal hearts were acutely perfused with a gap junction uncoupler, carbenoxolone (0-50µM) and the other 10 had patchy fibrosis induced with previous cardiac surgery.

Myocardial Infarction Surgery

10 out of 13 rats survived myocardial infarction surgery, the three death were related to peri-procedural arrhythmia. 10 Sprague Dawley rats were anaesthetized with 5% isoflurane in an induction chamber and intubated. The rats were ventilated using a Harvard rodent ventilator. Carprofen (5mg/kg), Enrofloxacin (5mg/kg), Marcaine (0.5%) was administered. The chest wall was cleaned using betadine and draped. A left sided thoracotomy was performed at the level of the 4th intercostal space with a small oblique incision. The subcutaneous layer was blunt dissected, pectoralis major and minor retracted to expose the intercostal muscles. A small incision was then made in the 4th intercostal space and the heart was exposed. The pericardium was gently stripped and the thymus was clamped in position to stabilise the heart. A small slip-knot suture was placed around the left anterior descending artery for 20 minutes using Prolene 7-0 around 3mm below the lower border of the left atrium at a depth of 1-2mm. Infarction was confirmed by pallor and akinesis of the anterior wall of the LV and apex. The slip knot was then released and reperfusion confirmed with visual inspection of the LV wall. The rats were extubated and recovered in a warm chamber until ambulant. The infarcts were matured for four weeks prior to the experiment.

Langendorff Perfusion

All eighteen explanted hearts were heparinized, and rapidly perfused ex-vivo on a Langendorff apparatus with Krebs-Henseleit solution (in mmol/l: NaCl 118.5, CaCl2 1.85, KCl 4.5, glucose 11.1, NaHCO3 25, MgSO4 2.5, NaH2PO4 1.4) gassed with 95% O2/5% CO2 at 37 °C ± 0.5 °C and pH 7.35 ± 0.05. A 10 minute stabilisation period was allowed during which the flow rate (10-15ml/min), temperature (37 °C ± 0.5 °C) and perfusion pressure through the aorta was maintained between 90 and 100 mmHg. In 8 of the hearts carbenoxolone (0-50µM) was added to

the perfusate and infused for at least 10 minutes prior to arrhythmia provocation. VF was induced with provoked electrical stimulation using a burst pacing protocol and sustained with pinacidil (30μM). Criteria for exclusion from the study was a heart rate less than 300 and spontaneous ventricular arrhythmia during the stabilization phase prior to VF optical mapping.

Programmed Electrical Stimulation (PES)

PES was carried out with silver electrodes placed at the bases of the left ventricles with a MicroPace system (Micropace EP, Santa Ana, USA). A burst pacing protocol (2mA, cycle length 50-70ms, 30 beat train) was used to induce VF. All hearts were pre-treated with a potassium channel opener, Pinacidil (30μM) during the stabilisation period described above to aid maintenance of VF prior to optical mapping studies.

Organisational analysis

The processed optical mapping data were firstly analysed to quantify the degree of global organisation with two novel independent methods, frequency dominance index (FDI) and causality pairing index (CPI)

Frequency dominance index

The frequency dominance index (FDI) calculates the total level of organisation by analysing all the dominant frequencies within a fibrillating ventricle. The FDI is defined as the proportion of the mapped area in fibrillation occupied by the highest amplitude dominant frequency in the global spectrum relative to the area of all frequencies in the mapped area. It is expressed as a

proportion value between 0 to 1. The methodology for calculating dominant frequency has been previously described in detail [2]. For instance, a dominant frequency signal of 14 Hz in 60% of the mapped areas would give a FDI of 0.6.

Causality Pairing index (CPI)

The mathematical details of calculating CPI have been described in detail below in the section titled *Granger causality*. The CPI calculates organisation of fibrillation from Granger causality (GC) analysis. GC is a measure of temporal dependency and it has been widely used in multivariate time series study [3–5]. In our study, the temporal dependence structure between signals from different pixels was inferred from fitting a vector auto-regression model to a multi-variate signal (A_τ, described below). Thereafter, the causality pairing index (CPI) was measured by quantifying the percentage of possible pixel pairings between which there is a propagational effects on a normalized scale of 0 to 1, where 0 is defined as no possible pairing having causal dependency and 1 where all possible pairings have causal dependency. The more stable the propagational effects between the pixel pairings, the more organised the data. After quantifying the level of global organisation in the VF data with FDI and CPI analysis in 50% of full resolution, we repeated the analysis at 25% and 12.5% of full spatial resolution and compared whether there was a correlation with different measures of stability and organisation (n_r/l_r and l_{ps}) calculated from full resolution phase analysis data described below.

Phase Mapping

After quantifying the level of global organisation of VF with FDI and CPI analysis, we characterised the underlying mechanism using phase analysis and quantified rotational drivers and their characteristics. Our methods for phase analysis and tracking of rotational drivers have previously been described in detail [2,6]. Propagation of activating wavefront in fibrillation is inherently complex, with multiple competing wavefronts moving in different direction and multiple deflections in signals with low spatiotemporal stability. Phase mapping is a technique that captures wavefront dynamics by annotating fluorescence or voltage data over time to a format that captures activation-recovery of tissue, elucidates periodicity, more clearly identifies wavefronts and localises PS points. Briefly, each pixel of optical fluorescence data was tagged for the minima and maxima and filtered to remove small amplitude fluctuations in the signals and fitted to a cubic spline to subtract the average of the minima and maximas splines to generate a zero mean. The real and imaginary parts of the Hilbert transform of this zero-mean signal were plotted in the phase plane and the phase angle calculated from this. A phase map of VF at each sampled time point was constructed and PS tagged using our algorithm. The edge of each wavefront was tracked in a 9x9 pixel window and maximum number of rotations [max (n_{rr})] calculated. A minimum 2 rotation filter was used to threshold and define a significant rotational driver and to construct phase singularity / rotational driver heats maps (incidence/second) from full resolution data for validating our methodology for fibrillation analysis.

Phase characterisation of organisation and stability

From phase processed fibrillatory data, rotational activity was quantified by our metrics of organisation and stability (n_r/l_r and l_{ps}) and compared with CPI, FDI and a more widely used analysis feature in fibrillation literature, Shannon entropy (Sh_{en}). Phase singularities with ≥2

rotations were labelled ‘significant’ rotations drivers (n_r) and the number of locations (pixels) they occupied (l_r) over a fibrillatory recording was tracked, thus t_r divided by l_r acted as a measure of stability and organisation, whereby rotational drivers with high number of rotations localising to a small area would generate the highest values by this metric. Phase singularities with <2 rotations with labelled ‘non-significant’ phase singularities and the number of locations (pixels) they occupied (l_{ps}) acted as a measure of disorganisation and instability, whereby a large number of short lived meandering phase singularities would generate the highest value by this metric. A linear regression with these objective measures (n_r/l_r and l_{ps}) as response variables and FDI and causality pairing index (CPI) as explanatory variables was then performed. The same regression analysis was performed Shannon entropy (Sh_{en}), as the explanatory variable for the purpose of comparison. The methodology for Sh_{en} has previously been described in detail by us [2].

Granger Causality

Granger causality is a measure of temporal dependency and it has been widely used in multivariate time series study [3–5]. GC could be inferred by fitting a vector auto-regression model to a multi-variate signal, i.e.,

$$A_\tau = \arg\min \sum_{t=p+1}^{n_t} \left\| x(t) - \sum_{\tau}^{p} A_\tau x(t-\tau) \right\|_2 + \lambda \sum_{\tau}^{p} \left\| A_\tau \right\|_1 \quad (Equation\ 1)$$

where $x(t)$ is the multi-variate signal, A_τ the auto-regression coefficient matrix, τ is the time lag, p the maximal time lag of the model and λ the regularization coefficient. With the l_1 -norm

based regularization term $\sum_{\tau}^{p}||A_{\tau}||_1$, solving *Equation* 1 yields a more sparse and robust GC estimation A_{τ} [7].

In this work, the auto-regression model in *Equation 1* is fitted to optical mapping data $x(t)$, each row of which is the signal from one pixel. The Forward Backward Lasso GC is applied to obtain A_{τ} [8]. $p = 1$ to reduce the computation complexity, and our results show that $p = 1$ is enough to locate the driver. The temporal dependence structure between signals from different pixels could be inferred from A_{τ}: the element of the i -th row and j -th column in A_{τ} reflects the strength of the j -th row of $x(t)$ in predicting i -th row of $x(t)$. In other words, if the element i -th row and j -th column in A_{τ} is larger than 0, the propagation direction is from j -th row to the i -th row of $x(t)$.

Based on A_{τ}, a **causality pairing index (CPI)** is calculated as the normalised number of non-zero elements of A_{τ}, i.e.,

$$CPI = \frac{|\{A_{\tau}(i,j), A_{\tau}(i,j) \neq 0\}|}{n_p^2 - n_p} \text{ for } i,j = 1 \dots n_p \ \tau = 1 \dots p \quad (\textit{Equation 2})$$

Given the optical mapping data of n_p pixels, n_p^2 is the number of all the possible paring combinations of pixels. Thus**,** CPI measures the percentage of the pairings between which there is a propagational effects. We propose that the higher the number, the more stable propagational effects between the data, the more organised the data.

In order to localise the rotational activity, for each pixel i, the major source vector $\overleftarrow{v}_{i,i_s}$ is determined as the vector pointing pixel i from its major sources pixel i_s, i.e.,

$i_s = \arg\max\ A_\tau(i, i_s)$ for $i_s = 1 \ldots n_p$ and $A_\tau(i, i_s) \neq 0$ (*Equation 3*)

By plotting the all the major source vectors $\overleftarrow{v}_{i,i_s}$, a GC vector map could be derived, where the circulatory propagation directions could be identified around the rotational driver. A local GC map could be derived in a similar manner, and a **circular interdependence value (CIV)** is calculated as:

$$CIV = \sum_{n_c=1,2} \frac{\sum_i \text{sign}(\overleftarrow{v}_{i,i_s} \times \overleftarrow{v}_{i,0})}{|\{i\}|} \text{ for } ||\overleftarrow{v}_{i,0}|| < \sqrt{2} d n_c \text{ (Equation 4)}$$

Where $\overleftarrow{v}_{i,0}$ is the vector pointing from the centre of the down-sampled area to pixel i and d is the interpixel distance (described below). With sign($\overleftarrow{v}_{i,i_s} \times \overleftarrow{v}_{i,0}$) as the direction of the cross product of the major source vectors $\overleftarrow{v}_{i,i_s}$ and $\overleftarrow{v}_{i,0}$, CIV measures the percentage of the major source vectors $\overleftarrow{v}_{i,i_s}$pointing to the same rotational direction, i.e., counter-clockwise or clockwise. CIV = 1 indicates that all the major source vectors $\overleftarrow{v}_{i,i_s}$ surrounding the centre of the down-sampled area are of the same rotational direction, and subsequently, the center is highly likely to be the driving area.

Down-sampling to lower spatial resolution for GC vector mapping.

In this work, we down sampled all optical mapping data to 25% of full spatial resolution in a grid of 8-by-8 points for GC vector mapping, whereby the spatial distance between two neighbouring (d) = 4 pixels. A larger interpixel distance of 8 corresponding to 12.5% spatial resolution subtended nearly the entire width of mapped area and corresponding GC vector maps were too sparse and not interpretable at this resolution.

Receiver operator curve of RD prediction based on CIV

The threshold of CIV is determined by RD prediction using CIV based on rat VF model. The whole rat heart was evenly sampled by the 4-by-4 squares along the long and short sides of the the optical mapping data with 50% overlapping, and subsequently, the distance between the centers of the two neighbouring sampling square is 8 pixels, approximately 8.75 mm. Given the phase mapping results, stable rotational sites are defined as locations where rotational activities stayed for more than 3 full rotations. Square sample areas are labelled as rotational if its center is a stable rotational site, and labelled as non-rotational otherwise. For each down-sampled square area, CIV was obtained upon applying GC mapping. With the labelling using high-resolution phase mapping, a receiver operator curve (ROC) for RD prediction using CIV was plotted as shown below. The optimal ROC operating points is 0.61, which is used as the threshold for defining RD positive sites.

Histology

5 out of 10 ischaemia-reperfusion hearts underwent histology after optical mapping studies. The samples were fixed in 4% formaldehyde and thereafter embedded in paraffin-wax. 10 µm thickness sections were cut at intervals of 1mm from the apex to the base of the ventricles and stained with Picrosirus red stain (Abcam, Cambridge, UK) for characterizing fibrosis. . Digital images were acquired with high-resolution scanning of slides using a widefield HWF1 Zeiss AxioObserver microscope. The images were scanned using the Zeiss proprietary Zen2012 Acquisition software (Carl Zeiss AG, Germany). The images were analysed in Fiji64 software (ImageJ, open-source).

Human VF mapping

Our GC-based analysis tools were tested on representative human VF optical mapping data that was previously acquired as part of a separate study by Aras et al. (2018) and the methodology reported in detail [1]. Donor human hearts were arrested using ice-cold cardioplegic solution and explanted in the operating theatre and transported to a laboratory for electrophysiology studies. Briefly, we tested our GC-based analysis tools on 33 VF recordings from 12 representative de-identified human donor hearts. These recordings were 4-seconds in duration and taken from coronary perfused LV wedge preparations that had VF induced with 25µM pinacidil pre-treatment. The mean LV wedge dimensions were 7 cm x 3.5 cm x 1.8 cm (height x width x thickness, equating to 44.1cm3). The critical wavelength volume for sustaining fibrillation was calculated to be as low as 7.4 cm^3 in some hearts. The wavelength volume was determined from the formula wavelength volume (Vλ) =λL×λTV×λTM wavelengths in longitudinal (λL) x transverse (λTV) x transmural (λTM) plane by Aras et al. (2018) [1]. VF was sustained where tissue volume/Vλ was above a safety factor κ=4.4±0.6, which effectively translates to an effective minimal tissue volume of 10 cm^3 for sustained VF (23% that of the mean LV wedge volume in this work).

Clinical AF mapping

In 16 patients presenting with symptomatic persistent AF for a first ablation procedure, electrograms were acquired using a 20-pole double-loop catheter (InquiryTM AFocusIIT^{M}, St Jude Medical, MN, USA) with 4mm electrode spacing. The term 'kernel' defined an area or location of atrial myocardium mapped that is subtended by the AFocusII^{TM} mapping catheter. The data was imported from Ensite^{TM} Velocity into MATLAB R2018 (MathWorks, Massachusetts, USA) using a custom made script. 20-seconds of bipolar electrogram data were

processed with band-pass (40-250Hz) and low-pass filtering (with a cut off <25Hz) and followed by signal rectification. The entire recording was used for organisational analysis with CPI. GC-vectors were plotted for each kernel and the CIV calculate to localise RDs. CIV threshold for localising RDs was established as 0.61 by plotting rat VF data on a receiver operating characteristic curve. To allow for meandering of RDs temporally, 8-second windows with overlapping window-shifts of 1-second was applied to segment the electrogram data.

Statistical Analysis

All statistical analysis was performed using a statistics software package (Prism version 5, Graphpad Software, California, USA) or MATLAB. After normality testing, student t tests were used to compare means between two groups. For each optical mapping recording, objective measures derived from optical mapping analysis were calculated, together with FDI, CPI and Sh_{en}. Linear regression models were fitted to FDI, CPI or Sh_{en} as explanatory variables and l_{ps} or nr/l_r as response variables, and F-test was applied for the linear models. R-squared measures were applied to test the strength of the relationship between the model and the dependent variable. $P < 0.05$ was regarded as significant. Results are expressed as mean ± SEM.

Supplementary figures and figure legends.

Supplementary Figure 1. Patchy ventricular fibrosis generated with ischemia- reperfusion. Representative histology of left ventricular slices axial slices from ischaemia-reperfusion infarcted hearts showing fibrosis (red to dark red) and normal ventricular tissue (orange to pink) from the apex (left) to the base (right). *(Abbreviations LV – Left Ventricle, RV- Right Ventricle)*

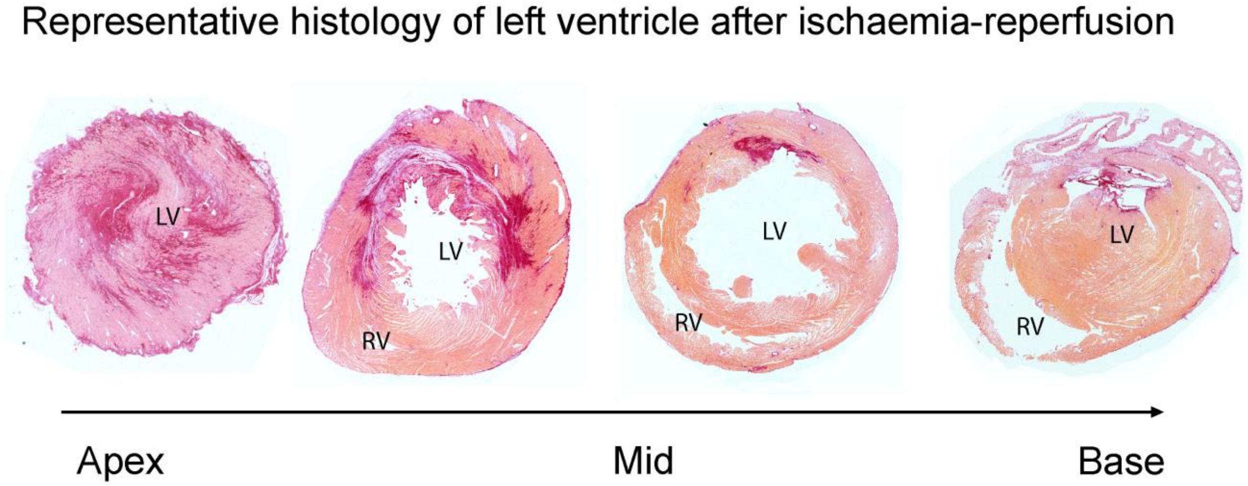

Supplementary Figure 2. Frequency dominance index (FDI) and causality pairing index (CPI) can characterise the global organization of fibrillation at high to low spatial resolution. Graphs showing positive correlation between a measure of organisation and stability – number of stable rotational drivers with >2 rotations (n_r) divided by number of locations and pixels they occupy (l_r) and CPI **(A)** and n_r/l_r and FDI **(B),** and no correlation between n_r/l_r and shannon entropy (Sh_{en}) **(C),** at decreasing resolutions of 50% (left), 25% (middle) and 12.5% (right) of full resolution from optical mapping of ventricular fibrillation (VF). (*Linear regression analysis, F-Test, coefficients of determination - R^2 and p values are indicated, n =18*)

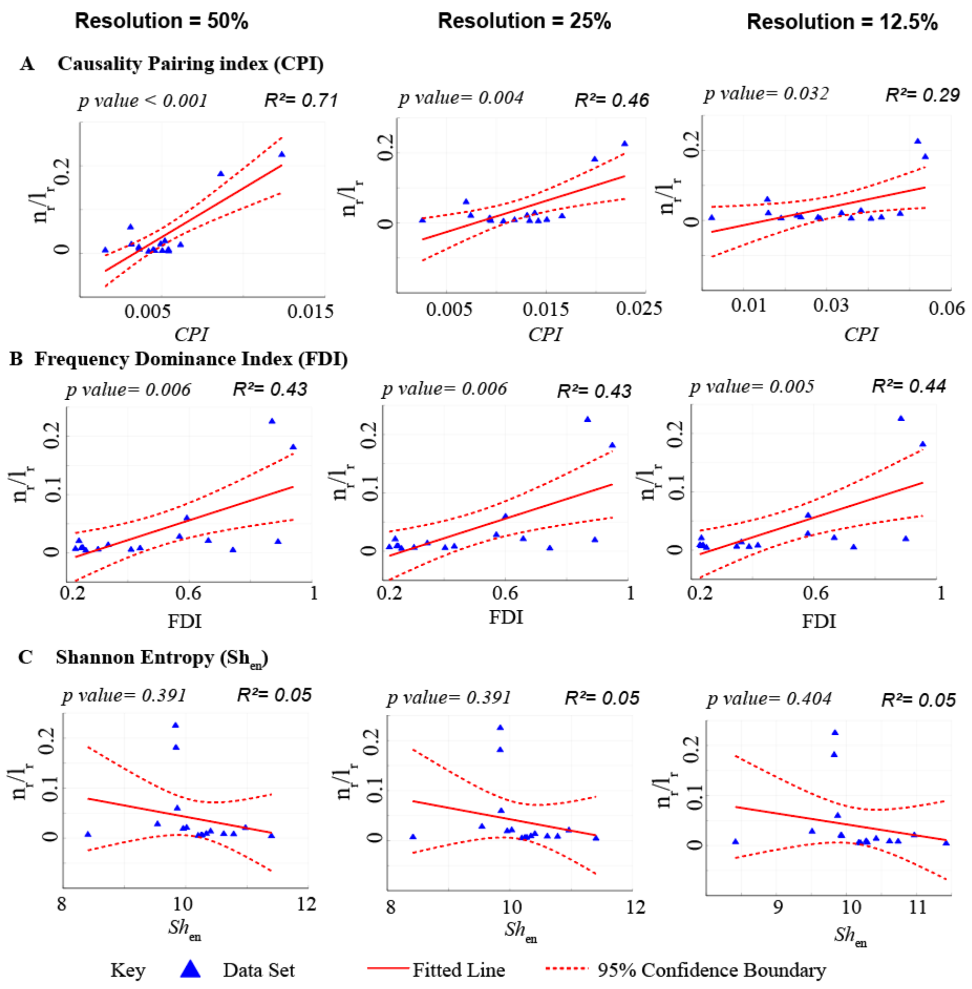

<u>Supplementary Figure 3</u>. Receiver operator curve (ROC) to determine the threshold value for a RD positive site from CIV values of RD positive sites across all the hearts across multiple sampled sites.

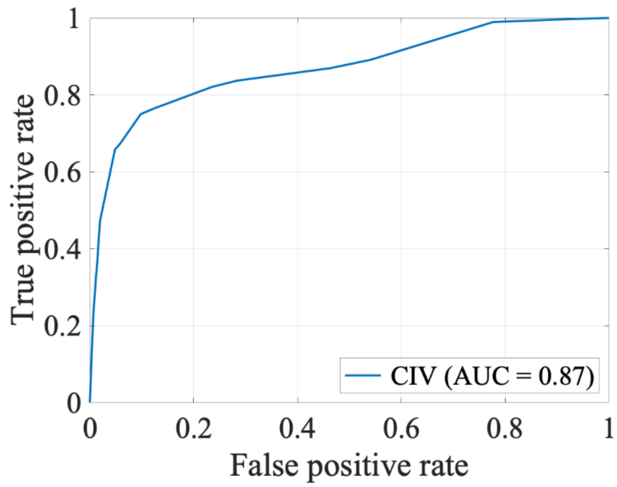

References

1. Aras KK, Faye NR, Cathey B, Efimov IR. Critical Volume of Human Myocardium Necessary to Maintain Ventricular Fibrillation. Circ Arrhythm Electrophysiol. 2018;11:e006692.

2. Handa BS, Roney CH, Houston C, Qureshi NA, Li X, Pitcher DS, Chowdhury RA, Lim PB, Dupont E, Niederer SA et al. Analytical approaches for myocardial fibrillation signals. Comput Biol Med. 2018;102:315–326.

3. Granger CWJ. Investigating Causal Relations by Econometric Models and Cross-spectral Methods. Econometrica. 1969;37:424–438.

4. Granger CWJ. Testing for causality: A personal viewpoint. J Econ Dyn Control. 1980;2:329–352.

5. Silvapulle P, Choi J-S. Testing for linear and nonlinear granger causality in the stock price-volume relation: Korean evidence. Q Rev Econ Financ. 1999;39:59–76.

6. Roney CH, Cantwell CD, Qureshi NA, Chowdhury RA, Dupont E, Lim PB, Vigmond EJ, Tweedy JH, Ng FS, Peters NS. Rotor Tracking Using Phase of Electrograms Recorded During Atrial Fibrillation. Ann Biomed Eng. 2017;45:910–923.

7. Arnold A, Liu Y., Abe N. Temporal causal modeling with graphical granger methods. Proc. 13th ACM SIGKDD International Conference on Knowledge Discovery and Data Mining. 2007;66-75.

8. Cheng D, Bahadori MT, Liu Y. FBLG: A Simple and Effective Approach for Temporal

Dependence Discovery from Time Series Data. 20th ACM SIGKDD Int Conf Knowl Discov Data Min. 2014;382–391.
