# Supplementary material for: Granger Causality–Based Analysis for Classification of Fibrillation Mechanisms and Localization of Rotational Drivers
Source: Circ Arrhythm Electrophysiol. 2020 Feb 16;13(3):e008237. doi: 10.1161/CIRCEP.119.008237 (PMC7069398; doi:10.1161/CIRCEP.119.008237)
Supplement: Supplementary file 2 [file hae-13-e008237-s002.pdf]

Granger Causality-based Analysis for Classification of Fibrillation Mechanisms and Localisation of Rotational Drivers

Running title: *Handa et al.; Granger Causality-based fibrillation analysis*

Balvinder S. Handa, BSc, MRCP[1]; Xinyang Li, PhD[1]; Kedar K. Aras, PhD[2]; Norman A. Qureshi, MRCP, PhD[1]; Ian Mann, MRCP[1]; Rasheda A. Chowdhury, PhD[1]; Zachary I. Whinnett, BM, BS, PhD[1]; Nick W.F. Linton, MBBS, PhD[1]; Phang Boon Lim, MB, BCHIR, PhD[1]; Prapa Kanagaratnam, FRCP, PhD[1]; Igor R. Efimov, PhD[2]; Nicholas S. Peters, FRCP, MD, FHRS[1]; Fu Siong Ng, MRCP, PhD[1]

[1]National Heart & Lung Institute, Imperial College London, United Kingdom; [2]Department of Biomedical Engineering, George Washington University, Washington, DC

American Heart Association.

Circulation Arrhythmia and Electrophysiology

Correspondence:

Dr Fu Siong Ng,

4th Floor, Imperial Centre for Translational and Experimental Medicine,

Imperial College London, Hammersmith Campus,

Du Cane Road

London W12 0NN

United Kingdom

E-mail: f.ng@imperial.ac.uk

Journal Subject Terms: Ventricular Fibrillation; Atrial Fibrillation; Basic Science Research; Mechanisms; Translational Studies

Abstract

Background - The mechanisms sustaining myocardial fibrillation remain disputed, partly due to a lack of mapping tools that can accurately identify the mechanism with low spatial resolution clinical recordings. Granger causality (GC) analysis, an econometric tool for quantifying causal relationships between complex time-series, was developed as a novel fibrillation mapping tool and adapted to low spatial resolution sequentially-acquired data.

Methods - VF optical mapping was performed in Langendorff-perfused Sprague-Dawley rat hearts (n=18), where novel algorithms were developed using GC-based analysis to: a) quantify causal dependence of neighbouring signals and plot GC-vectors, b) quantify global organisation with the causality pairing index (CPI), a measure of neighbouring causal signal pairs and c) localise rotational drivers (RDs) by quantifying the circular interdependence of neighbouring signals with the circular interdependence value (CIV). GC-based mapping tools were optimised for low spatial resolution from down-sampled optical mapping data, validated against high-resolution phase analysis and further tested in previous VF optical mapping recordings of coronary perfused donor heart LV wedge preparations (n=12), and adapted for sequentially-acquired intracardiac-electrograms during human persistent atrial fibrillation (PsAF) mapping (n=16).

Results - Global VF organisation quantified by CPI showed a negative correlation at progressively lower resolutions (50% resolution: p=0.006, R^2=0.38, 12.5% resolution, p=0.004, R^2=0.41) with a phase analysis derived measure of disorganisation, lps. In organised VF with high CPI values, GC-vector mapping characterised dominant propagating patterns and localised stable RDs, with the CIV showing a significant difference in driver versus non-driver regions (0.91±0.05 vs 0.35±0.06, p=0.0002). These findings were further confirmed in human VF. In PsAF, a positive correlation was found between the CPI and presence of stable RDs (p=0.0005,R^2 =0.56). 50% of patients had RDs, with a low incidence of 0.9±0.3 RDs/patient.

Conclusions - GC-based fibrillation analysis can measure global fibrillation organisation, characterise dominant propagating patterns and map RDs using low spatial resolution sequentially-acquired data.

Keywords: ventricular fibrillation; atrial fibrillation; Granger causality; rotational drivers; causality pairing index

American Heart Association
Circulation
Arrhythmia and Electrophysiology

Non-standard Abbreviations and Acronyms:

AF – atrial fibrillation

CIV – circular interdependence value

CMOS - complementary metal oxide semiconductor

CPI – causality pairing index

DF – dominant frequency

DMSO – dimethyl sulfoxide

ECGI - electrocardiographic imaging

EGMs – electrograms

FDI – frequency dominance index

GC – Granger causality

L_{ps} –locations occupied by phase singularities

L_r – locations occupied by a rotational driver

LV – left ventricle

N_r – number of rotations

OHS – open heart surgery

PES - programmed electrical stimulation

PS – phase singularity

Sh_{en} – Shannon entropy

RD- rotational driver

STAR - Stochastic Trajectory of Ranked Signals

VF – ventricular fibrillation

VT – ventricular tachycardia

American Heart Association
Circulation
Arrhythmia and Electrophysiology

Background

The underlying mechanisms sustaining myocardial fibrillation remain unclear and there is a lack of consensus on a unifying mechanism. The anarchical hypothesis of multiple self-propagating

wavelets and the opposing hierarchal hypothesis of organised spiral wave re-entry, referred to as rotors or rotational drivers (RDs), organised around a non-anatomical unexcited core termed a phase singularity (PS) point, continue to be debated as the mechanism sustaining fibrillation. Although evidence exists from high spatiotemporal resolution optical mapping studies to support these conflicting mechanisms [1–4], clinical translation to guide treatment remains challenging due to the poor spatiotemporal resolution of clinical mapping systems and a lack of suitable analysis tools. Thus, outcomes from catheter ablation remain poor, especially in persistent atrial fibrillation (AF), where recurrence remains as high as 40-50% [5].

Multiple prominent investigators have proposed that ablation of sites localising RDs can terminate AF [6] and prevent ventricular fibrillation (VF) reoccurrence in survivors of sudden cardiac death [7]. Others have found no evidence for their existence in fibrillation mapping studies during cardiac surgery with high resolution electrode arrays [8] and proposed complex asynchronous endocardial-epicardial disassociation of fibrillatory conduction as a further mechanism.

American Heart Association.
Circulation
Arrhythmia and Electrophysiology

A challenge in mapping fibrillatory mechanism is its dynamic nature. There is beat-to-beat variability in periodicity and amplitude of signals, and global wavefront propagation is non-uniform and temporally variable. RDs often demonstrate meandering and transient trajectories [9]. Conventional mapping techniques, such as activation mapping which require annotation of a reference signal [10] and stable linear propagation are poorly suited. To overcome this issue, phase analysis is frequently used in fibrillation mapping instead. Phase analysis assigns a phase value between π and $-\pi$ to the activation-recovery cycles of a given myocardial area [11]. Tracking these changes in phase allow for annotation of propagating wavefronts and localisation of PSs as areas devoid of a definitive phase. Accurate phase analysis however requires both global panoramic

mapping and adequate spatial resolution [12], which is currently not possible with clinically-available tools.

We previously demonstrated that RD localisation from phase analysis of intracardiac electrograms (EGMs) acquired by multipolar catheters, including the 64-electrode basket catheters is inaccurate as it lacks sufficient resolution, requires significant interpolation, stitching of sequentially acquired data and is prone to generation of a high number of false positive RDs [13]. Other research groups have also highlighted the limitations of phase analysis with low spatial resolution [14] and demonstrated an increase in false positive RD detection rate with increasing inter-electrode distance and noise [15]. In addition, basket catheters can provide incomplete surface coverage and are susceptible to poor contact [16]. Alternatively, global cardiac mapping non-invasively is possible from the body surface using a multielectrode electrocardiographic imaging (ECGI) vest. ECGI utilises inverse solution algorithms for interpolating intracardiac EGMs. Whilst there are some limitations with ECGI, such as correlation between surface and contact EGMs [17] and noise artefact, it does provide higher resolution mapping and has shown potential in a number of early studies where it has been utilised to map AF [18], VF [7] and VT [19] mechanisms.

American Heart Association.
Circulation
Arrhythmia and Electrophysiology

Here, we propose that granger causality (GC) analysis, originally an econometric tool designed to determine causal relationships between complex time series data [20], can be repurposed as a novel tool to analyse fibrillation. Given GC analysis depends only on neighbouring causal relationships, we postulated that it could overcome the limitation of spatial resolution and sequentially acquired limited coverage data in fibrillation. We generated a range of fibrillation mechanisms in a rat VF model by modulating gap junction coupling and fibrosis, two factors implicated in cardiac remodelling. We hypothesised that GC-based analysis can be used to: (a) analyse temporal dependence of fibrillatory signals in neighbouring regions and

determine the dominant propagating pattern, (b) quantify the global organisation and general mechanism of fibrillation, and (c) map stable RDs at low spatial resolution with limited coverage. Initially developed and validated against high-resolution phase analysis in a rat VF model, these novel GC-based analysis tools were further tested in previous VF optical mapping recordings of coronary perfused donor heart LV wedge preparations and finally adapted to analyse multi-electrode catheter recordings of persistent AF patients.

Methods

The data, analytic methods, and study materials are available from the corresponding author to other researchers for the purposes of reproducing the results or replicating the procedure upon reasonable request. Methods are described briefly here. For full details, please see the supplemental materials.

American Heart Association. Circulation Arrhythmia and Electrophysiology

Ethical Approval

The animal work was performed in accordance with standards set out in the United Kingdom Animals (Scientific Procedures) Act 1986 and was approved by Imperial College London Ethical Review Board under the project license PEE7C76CD and PCA5EE967. For the clinical component of the study, patients with symptomatic persistent AF presenting for their first ablation to Imperial College Healthcare NHS Trust were prospectively enrolled. The study was approved by the Local Research and Ethics Committee for Imperial College Healthcare NHS Trust and written informed consent were obtained from all patients. Experiments using human heart tissue were previously approved by the Institutional Review Board (Office of Human Research) at the George Washington University [21].

Experimental Protocols

Eighteen Sprague-Dawley rats (250-300g) were humanely killed and the hearts were explanted, heparinised, and rapidly perfused ex-vivo on a Langendorff apparatus with Krebs-Henseleit solution, and stabilised for a 15 minute period before *ex vivo* optical mapping studies of transmembrane potential. In order to create a range of VF activity, in group one 8 of the hearts were acutely perfused with a gap junction uncoupler, carbenoxolone (0-50μM), which in our previous experiments produced increasing disorganised VF at increasing doses. In group two, the other 10 hearts had chronic patchy ventricular fibrosis which had been induced with ischaemia-reperfusion cardiac surgery four weeks prior to the experiment **(Supplementary Figure 1)**. No drugs were added to the perfusate, and in our previous experiments patchy fibrosis was found to sustain a more organised form of VF. Programmed electrical stimulation (PES) using a burst pacing protocol with the aid of Pinacidil 30μM was used to induce and sustain VF.

Optical mapping

Explanted hearts underwent optical mapping of the left ventricular anterior wall after VF induction. The transmembrane voltage was recorded from optical mapping fluorescence data using our custom made complementary metal oxide semiconductor (CMOS) camera (Cairns, Feversham UK) utilising the potentiometric dye RH237 (25μl of 1mg/ml dimethyl sulfoxide [DMSO]; Thermo-Fisher, Massachusetts, USA) and excitation-contraction uncoupler blebbistatin (10μmol/L, Tocris Bio-Sciences, Cambridge UK) in 160 x 128 pixel resolution for a 10 second duration. All our methods for filtering and analysing optical mapping fluorescence data have been previously described in detail [22,23].

American Heart Association. Circulation Arrhythmia and Electrophysiology

Organisational analysis

The processed optical mapping data were firstly analysed to quantify the degree of global organisation with two novel independent methods; causality pairing index (CPI) derived from global GC analysis and frequency dominance index (FDI) derived from the dominant frequency (DF) analysis. These indices are described below in detail.

Granger causality analysis

GC analysis is an econometric methodology for quantifying the causal dependence between two or more complex time series using a linear autoregressive model [20,24,25]. GC is a concept based on statistical prediction, whereby GC analysis statistically tests if a given time series signal A causes time series signal B, by analysing information contained in past values of time series A and determining if the information contained in signal A can predict signal B, beyond predictions from past values of signal B alone. In this work, we developed novel GC-based tools for fibrillation analysis adapted for low spatial resolution and limited coverage sequential mapping from optical fluorescence data of transmembrane potentials in a perfused rat VF model. GC analysis was used to measure the strength of the causal relationship between signals in neighbouring regions, and to quantify whether the fibrillation signal in one region over time could predict signals in another. GC vector mapping (described below) was performed based on quantified strengths of these relationships to determine the dominant propagating patterns.

Causality pairing index

CPI calculates global organisation of fibrillation from GC analysis, as described above. In this study, the temporal dependence structure between signals from different pixels was calculated by fitting a vector auto-regression model to a multi-variate signal. Thereafter, the CPI was measured by quantifying the percentage of possible pixel pairings between which there are propagational

American Heart Association. Circulation Arrhythmia and Electrophysiology

effects on a normalised scale of 0 to 1, where 0 is defined as no possible pairing having causal dependency and 1 where all possible pairings have causal dependency **(Figure 1A, 1B)**. The more pixel pairings that have a propagational effect between them above a specified threshold, the greater the level of global organisation in fibrillation.

Frequency dominance index

The FDI calculates the total level of global organisation by analysing all the DFs from all the signals within a fibrillating ventricle. The FDI is defined as the proportion of area in fibrillation occupied by the highest amplitude DF in the global spectrum relative to the area of all frequencies in the mapped area **(Figure 1C)**. The methodology for calculating DF has been previously described in detail [26].

American Heart Association

Circulation

Arrhythmia and Electrophysiology

Down-sampling spatial resolution

After quantifying the level of global organisation in the VF data with CPI and FDI, we tested whether these indices were reliable and adaptable to lower spatial resolutions. CPI and FDI were benchmarked at decreasing spatial resolution (with data down-sampling) against two measures of global fibrillatory organisation, l_{ps} and n_r/l_r, generated from full spatial resolution phase processed data.

Phase Mapping

Rat VF phase processed analysis from full spatial resolution optical mapping was used for fully characterising the underlying mechanism of fibrillation, and for benchmarking our novel GC-based tools. Our methods for phase analysis and tracking of RDs have previously been described in detail [26, 27]. A phase map of VF at each sampled time point was constructed and PS tagged using our algorithm. The edge of each wavefront was tracked in a 9x9 pixel window and maximum number of rotations [max (n_r)] calculated. A minimum 2-rotation filter was used to

threshold and define a significant RD and to construct RD heat maps from full spatial resolution data for validating our methodology for fibrillation analysis.

Phase characterisation of organisation and stability

From phase processed fibrillatory data, rotational activity was quantified by our metrics of organisation and stability (n_r/l_r and l_{ps}) and compared with CPI, FDI and a more widely used analysis feature in fibrillation literature, Shannon entropy (Sh_{en}) [28]. PS with < 2 were labelled 'non-significant' PSs and the number of locations (pixels) they occupied (l_{ps}) acted as a measure of global disorganisation, spatial meander and instability, whereby a large number of short lived meandering PSs would generate the highest value by this metric. PSs with ≥2 rotations were labelled 'significant' rotations drivers (n_r) and the number of locations (pixels) they occupied (l_r) over a fibrillatory recording was tracked, thus n_r divided by l_r acted as a measure of stability and global organisation, whereby RDs with high number of rotations localising to a small area would generate the highest values of this metric. These objective measures of fibrillation organisation (n_r/l_r and l_{ps}) calculated from high resolution phase analysis were correlated with our novel low resolution measures of fibrillation organisation (CPI and FDI). In addition, we correlated a conventional fibrillation analysis tool, Shannon entropy (Sh_{en}) [26,28], with CPI and FDI.

American Heart Association.
Circulation
Arrhythmia and Electrophysiology

GC Vector Maps

GC vector mapping was tested as a low spatial resolution tool adapted specifically for limited spatial coverage to determine dominant propagation and localise RDs independent of phase analysis. In this work, GC vector maps were generated from an 8 x 8 data grid at 25% of the full spatial resolution optical fluorescence data, whereby three pixels were discarded between each data point. Within this 8 x 8 data grid, an algorithm determined firstly if there were any signals with temporal causal dependence, thereafter it quantified the strength of the temporal causal

dependence between these signals. A vector was then plotted only between the source signal and the signal where it exerted the greatest causal or propagational effect above a specified threshold **(Figure 1A)**. GC vector maps were benchmarked against full resolution phase processed data to determine ability to characterise fibrillation mechanism.

A circular interdependence value (CIV) is proposed to quantify the circular interdependence of signals in the local 8 x 8 GC data grid and identify the location of a RDs. For each GC vector, we calculated the cross product relative to the centre of the grid using the right hand rule **(Figure 1D)**. The resultant cross product vector was binarised as either pointing up or down in a virtual three dimensional space. Using this principle, three possibilities exist: 1) an organised continuous circular one directional rotation of GC vectors over time would generate all virtual cross product 3D vectors in the same direction (either up or down) 2) GC vectors with a disorganised arrangement would generate cross product 3D vectors in both directions (up and down) dependent on degree of disorganisation 3) GC vector all in one direction, for instance a propagating linear wavefront, would also generate cross product 3D vectors in both direction. Thus by applying the equation below to these 3D vectors outputs the circular interdependence value can be calculated.

$$CIV = \left| \frac{No.\ of\ vector\ pointing\ \uparrow \text{(up)} -\ No.\ of\ \ vectors\ pointing\ \ \downarrow \text{(down)}}{Total\ number\ of\ vectors} \right|$$

CIV is quantified on a normalised scale of 0 to 1, whereby a stable RD present throughout the recording would generate a value of 1, and disorganised or propagating wavefronts in one direction will generate a value closer to 0.

***Ex vivo* human VF mapping**

Our GC-based analysis tools were tested on human VF optical mapping data that was previously acquired as part of a separate study by Aras et al. (2019) and the methodology was reported in

American Heart Association. Circulation Arrhythmia and Electrophysiology

detail [21]. Briefly, we tested our GC-based analysis tools on 33 VF recordings from 12 representative de-identified human donor hearts. These recordings were 4-seconds in duration and taken from coronary perfused LV wedge preparations that had VF induced with 25µM pinacidil pre-treatment. The mean LV wedge dimensions were 7 cm x 3.5 cm x 1.8 cm (height x width x thickness).

Clinical *in vivo* AF mapping

In 16 patients presenting with symptomatic persistent AF for a first ablation procedure, EGMs were acquired using a 20-pole double-loop catheter (InquiryTM AFocusII™, St Jude Medical, MN, USA) with 4mm electrode spacing. The term 'kernel' defined an area or location of atrial myocardium mapped that is subtended by the AFocusII mapping catheter. The data was imported from Ensite™ Velocity into MATLAB R2018 (MathWorks, Massachusetts, USA) using a custom made script. 20-seconds of bipolar EGM data were processed with band-pass (40-250Hz) and low-pass filtering (with a cut off <25Hz) and followed by signal rectification. The entire recording was used for organisational analysis with CPI. GC-vectors were plotted for each kernel and the CIV calculate to localise RDs. CIV threshold for localising RDs was established as 0.61 by plotting rat VF data on a receiver operating characteristic curve. To allow for meandering of RDs temporally, 8-second windows with overlapping window-shifts of 1-second was applied to segment the EGM data

Statistical Analysis

All statistical analysis was performed using a statistics software package (Prism vesion 5, Graphpad Software, California, USA) or MATLAB. After normality testing, student t tests were used to compare means between two groups. For each optical mapping recording, objective measures derived from optical mapping analysis were calculated, together with FDI, CPI and

American Heart Association. Circulation: Arrhythmia and Electrophysiology

Sh_{en}. Linear regression models were fitted to FDI, CPI or Sh_{en} as explanatory variables and l_{ps} or n_r/l_r as response variables, and F-test was applied for the linear models. R-squared measures were applied to test the strength of the relationship between the model and the dependent variable. $P< 0.05$ was regarded as significant. Results are expressed as mean ± standard error of mean (SEM).

Results

Fibrillation organisation quantified by the FDI and CPI

Rat VF optical fluorescence data were recorded in hearts with underlying chronic fibrosis, or acute GJ uncoupling with carbenoxolone. In previous experiments, with high-resolution phase processed data, a spectrum of fibrillatory mechanisms was found in these hearts, ranging from fibrillation driven by organised RDs to completely chaotic activity. At decreasing spatial resolutions, l_{ps}, a measure of global disorganisation correlated negatively with both CPI [50% resolution: $F(1,16)=9.9, p=0.006$, $R^2=0.38$, 25% resolution: $F(1,16) =11.7$, $p=0.004$, $R^2=0.42$ and 12.5% resolution $F(1,16)=11.0, p=0.004$, $R^2=0.41$] and FDI [50% resolution: $F(1,16)=10.4, p=0.005$, $R^2=0.39$, 25% resolution: $F(1,16) =10.4$, $p=0.005$, $R^2=0.39$ and 12.5% resolution $F(1,16)=9.8, p=0.006$, $R^2=0.38$] (**Figure 2A, 2B).** Disorganised fibrillation with a high number of meandering non-significant PSs had low FDI and CPI values. Conversely, n_r/l_r, a measure of global fibrillatory organisation that tracks presence of spatiotemporally stable RD from full resolution phase processed data, correlated positively with CPI and FDI at decreasing resolution **(Supplementary Figure 2A, 2B).** Shannon entropy values, more conventionally utilised in fibrillation analysis, showed no statistically significant correlation with l_{ps} or n_r/l_r **(Figure 2C, Supplementary Figure 2C).**

American Heart Association. Circulation Arrhythmia and Electrophysiology

After determining the applicability of our novel organisational indices to low resolution data, we selected representative hearts along the organisational spectrum, to delineate whether the fibrillatory mechanism characterised by full resolution phase analysis correlated with the level of global organisation as determined with CPI and FDI with low spatial resolution data. Hearts with the highest FDI had the most spatiotemporally stable RDs (**Figure 3A, 3B)**, with high numbers of rotations and much fewer short-lived PSs in comparison to hearts with a lower FDI **(Figure 3C)**. The RD heat map showed well localised discrete areas harbouring the organised RD in hearts with a high FDI, whereas hearts with a low to intermediate FDI values did not show such areas **(Figure 3A)**.

GC mapping to localise driver regions

American Heart Association
Circulation
Arrhythmia and Electrophysiology

After establishing that high global organisation in fibrillation, as measured by CPI and FDI, correlated positively with the existence of localised stable RDs, we tested whether GC vector mapping could identify causally dependent neighbouring regions to localise areas harbouring RDs at low spatial resolutions independent of phase analysis. In a representative heart with high global fibrillatory organisation, optical fluorescence data was down-sampled to 25% of full spatial resolution for GC vector mapping. GC vector mapping identified a number of regions with causal dependence and localised an area harbouring a spatiotemporally stable RD with a circular interdependence of GC-vectors as shown in **Figure 4A, 4B.** Optical fluorescence of the transmembrane potentials along this region showed repetitive sequential activation over time **(Figure 4C).** At 12.5% of the full spatial resolution RD regions could no longer be localised accurately with GC mapping (results not enclosed).

We further validated RD localisation with GC-vector mapping performed at 25% spatial resolution against full spatial resolution phase analysis in a further three hearts classified as

organised from high FDI and CPI measures. **Figure 5** shows GC-vector mapping from these hearts, where regions with circular interdependence of signals had high CIV values (1, 0.82, 0.9), and these regions highly correlated with regions localising stable RDs on full-resolution phase analysis. However, in non-driver regions where CIV values were low, GC vectors had a random or non-circular distribution, such as heart B area 2 (CIV= 0.35). Areas with meandering RDs with less stability showed intermediate organisation and an intermediate CIV value, such as heart A area 2 (CIV = 0.55, **Figure 5A)**. In regions harbouring stable RDs, the CIV was 2.6 fold higher than regions without RD (0.91±0.05 versus 0.35±0.06, p=0.0002, n=3, **Figure 5B**).

GC-based analysis of human VF

We further tested and validated our low spatial resolution adapted GC-based tools in optical mapping of human VF in LV wedge preparations to test applicability to a larger spatial scale and benchmarked these tools against full resolution phase-analysis. As with rat VF, a spectrum of VF mechanisms were found, ranging from fibrillation driven by organised RDs to completely chaotic activity. At decreasing spatial resolutions, l_{ps} correlated negatively with CPI as before; 50% spatial resolution: $F(1,10)=24.4$, $p<0.0001$, $R^2=0.42$ and 25% spatial resolution: $F(1,10) =21.3$, $p=0.0001$, $R^2=0.38$ **(Figure 6A)**. As before, the optical fluorescence data was downsampled to 25% of full spatial resolution for GC vector mapping. **Figure 6B** shows two representative hearts with low and high global organisation respectively. In a representative heart with a high global fibrillatory organisation, as quantified by CPI, GC vector mapping identified a regions with high CIV, harbouring a stable RD and this correlated with the same region identified by phase analysis on the RD heatmap. Similarly, in a representative heart with low global fibrillatory organisation, as quantified by CPI, GC vector mapping showed random vector

American Heart Association. Circulation Arrhythmia and Electrophysiology

distribution with low CIV values and this correlated with multiple wavelet driven fibrillation on phase analysis with no stable RDs.

GC vector mapping of intracardiac EGMs from AF mapping

After developing and validating these novel GC-based tools for use with low resolution data from optical mapping of transmembrane voltage, we adapted our methodology to process intracardiac EGMs in human persistent AF acquired sequentially using a 20-pole AFocusII^{TM} mapping catheter. We measured global fibrillatory organisation and localised RDs in persistent AF with our novel indices. Processing EGMs for GC analysis requires different considerations for signal processing than optical fluorescence data. Firstly, a three-dimensional spatial map was constructed from spatial correlates of the electrodes and corresponding bipoles. Each signal underwent high and low band pass filtering before been rectified and down-sampled. Causal dependence between bipoles was established from rectified down-sampled data **(Figure 7A). Figure 7B** demonstrates validation of this methodology with paced data with a wavefront emerging from near electrode 1,2 and propagating towards 7,8. If causal dependence between bipoles was present, it was shown with GC vectors (red arrows). The CIV of 0.11 for this representative paced data as expected is low.

American Heart Association.
Circulation
Arrhythmia and Electrophysiology

GC vector mapping quantifies global AF organisation and identifies areas harbouring RDs

The EGM recordings during AF mapping were processed and causality maps plotted from 20-seconds of recording. The threshold value for a RD positive site was determined from a receiver operating characteristic (ROC) curve **(Supplementary Figure 3)**. **Figure 8A** demonstrates a recording that was positive for a RD, where a circular interdependence of GC vectors can be seen between bipoles. The corresponding CIV value shows a small degree of fluctuation over time suggesting low meander and remains above the threshold value for a RD. The EGMs

demonstrate sequential activation between electrodes with causal dependence and rotational configuration over time **(Figure 8B, 8C)**. To the contrary, in sites testing negative for RDs **(Figure 8A-8C)** few electrodes demonstrate causal dependence and the vectors are randomly arranged. The CIV remains low and below the threshold for a RD throughout the recording period and the corresponding EGMs demonstrate chaotic activity with no discernible underlying patterns of activation.

To take into account the transient and meandering nature of RDs, the EGM recordings were segmented in 8-second overlapping windows in addition to analysis of the entire 20-second segment to detect RD positive sites and time windows The term "kernel" describes a recording set from an area subtended by a multipolar catheter. In the 16 patients with persistent AF, on average 14.8±1.5 kernels and 349.7±36.6 overlapping 8-second windows of recording were taken from each patient. Using GC-vector based identification of RDs, 50% of patients were found to have one or more RDs. Overall, persistent AF was found to be a largely disorganised rhythm, with a low incidence of stable RDs, with only 0.9±0.3 kernels and 1.8±0.7 8-second windows showing a RD per patient **(Figure 8D).** As seen in the *ex vivo* experiments above, the data demonstrates a spectrum of organisation underlying persistent AF as quantified by the CPI analysis. We found a positive correlation between the CPI values and presence of RDs [$F(1,14)= 20.3, p=0.0005, R^2$ 0.56, **Figure 8E].** In patients with low CPI fibrillation the underlying mechanism was chaotic activity with no RDs, whereas in patients with higher CPI values there was a higher number of RDs.

American Heart Association
Circulation
Arrhythmia and Electrophysiology

Discussion

In this study, we developed novel GC-based tools for fibrillation analysis adapted for use with low spatial resolution data acquired with limited coverage sequential mapping, independent of conventionally used phase analysis. We demonstrated that neighbouring regions in fibrillation demonstrate causal dependence, and that quantifying these causal relationships can determine global fibrillatory organisation using our novel indices such as CPI. We showed that high global fibrillatory organisation as measured with our novel indices CPI and FDI, positively correlated with existence of stable RDs. By interrogating causal relationships between neighbouring regions with GC vectors, we were able to show a continuous circular interdependence of GC vectors as the hallmark of areas harbouring stable RDs. We lastly developed a quantitative tool, the CIV, which could differentiate regions with high density of stable RDs activity, transient and meandering RDs and non-driver regions. These GC-based tools were developed for non-panoramic, low-resolution and sequential limited coverage mapping, validated against high-resolution phase analysis of rat VF and human VF optical mapping data and then adapted for use with EGMs from patients with persistent AF.

American Heart Association. Circulation Arrhythmia and Electrophysiology

Whilst certain triggers, such as ectopic firing from pulmonary veins in AF [29] and His-Purkinje system in VF [30] are well established, a key challenge in treating fibrillation is the difficulty in accurately determining the underlying fibrillatory mechanism and identifying putative drivers with clinical mapping systems that have substantially lower spatial resolutions than optical mapping and multielectrode array mapping used in pre-clinical research. This has led to conflicting data and multiple mechanism being implicated. The initial mathematical model of multiple self-propagating wavelets with no clear drivers, proposed by Moe et al.[31], has been supported by a number of pre-clinical and clinical studies [8,32,33]. More recently this hypothesis

has evolved with evidence supporting more complex three-dimensional mechanisms of asynchronous activation, connections and wavebreaks between the endocardium and epicardium in human AF mapped with high density electrode arrays during cardiac surgery [8,34,35]. However, some investigators continue to propose that there are regions of high spatiotemporal organisation driving fibrillation and cite acute termination of AF through targeted ablation of sites harbouring RDs as strong evidence for this hypothesis [36,37].

The clinically available fibrillation analysis tools, most widely available of which is phase analysis, considered to be the "gold-standard", have significant limitations. In AF simulation studies, we previously demonstrated that the spatial resolution of multiple commercially available clinical catheters including the AFocusII™ used in this study is prone to generating a large number of false positive RD detections from phase processed data and is ineffective at locating RDs with meandering trajectories. In perfused heart AF mapping studies, phantom RDs often appear as spatial resolution is reduced [38]. Phase mapping additionally requires careful consideration of a number parametric inputs; such as rotational thresholds for labelling RDs and average fibrillation cycle lengths for accurate analysis [39].

American Heart Association.
Circulation
Arrhythmia and Electrophysiology

GC-based analysis negates the issues generated by low spatial resolution phase analysis and dynamic nature of fibrillation by considering only causal interdependence of neighbouring areas over time rather than attempting to construct panoramic videos of wavefront dynamics within a chamber from interpolated, temporally stitched and sequentially acquired data to determine mechanism. Furthermore, by determining only causal relationships in neighbouring regions over long periods of mapping in fibrillation, where activation patterns are dynamic and vary beat to beat, GC mapping also addresses a key limitation of activation mapping in fibrillation, which is annotation of relative activation times from complex electrograms. GC

analysis was initially developed as an econometric tool, it has been used to characterise the causal connectivity between different brain regions based on neuroimaging data [40,41] and is widely used in climate science to establish causal relationships between two parameters [42].

By quantifying the circular interdependence of GC-vectors that characterises a RD with CIV through measurements of the directionality of cross-product vectors relative to the centre, we provide a non-subjective measure for localising RDs that does not rely on user interpretation. Windowing CIV in time segments can quantify the temporal stability of RDs, accounting for their transient and meandering nature and determine their overall contribution to the fibrillation mechanism.

We found a direct correlation between global fibrillatory organisation and the underlying mechanism in both rat VF and human VF, and therefore classifying this organisation with FDI or CPI in low spatial resolution without detailed mapping may help classify the predominant mechanism and guide treatment strategy. For instance, the operator may choose to pursue detailed mapping and RD ablation in patients with high FDI and CPI values only. Some clinical studies support the existence of a spectrum of organisation and mechanisms in fibrillation. For instance in VF mapping studies of patient undergoing cardiac surgery stable RDs, meandering RDs and multiple wavelets were all found as predominant mechanisms in different patients [43]. Similarly in AF, non-invasive mapping with ECGI has shown coexistence of a number of mechanisms and varying fibrillation complexity [18]. The global fibrillatory organisation and incidence of RDs were low in many of our persistent AF mapped patients. This suggests that mechanism guided ablation may only be suitable in a select number of persistent AF patients.

Whilst in our study we found evidence for existence of RDs with continuous circular organisation of GC-vectors in persistent AF, it is important to point out that a bi-atrial mapping

American Heart Association
Circulation
Arrhythmia and Electrophysiology

study of persistent AF patients using high resolution 512-electrode grid during open heart surgery (OHS) failed to show existence of stable RDs [44]. However in keeping with our findings, another similar study conducted during OHS with a 128-electrode grid, found a similar spectrum of mechanisms, ranging from disorganised activity through to transient RDs [45]. Both these studies only involved activation sequence analysis rather than a more sophisticated methodology specifically adapted for localising RDs. It is probable that RDs may not have been localised due to a lack of robust tools, such as the ones proposed in this work.

Other investigators have taken a similar approach to ours in characterising mechanisms by considering fibrillatory conduction as propagation within a communication network where neighbouring regions exert influence over each other over time. Quantifying and mapping this functional connectivity using mutual information analysis [46] has therefore been explored in AF, although in a differing context, whereby greater connectivity or organisation was found in patients with successful ablations. A parallel probabilistic analysis technique looking at proportion of time neighbouring signals precede each other called Stochastic Trajectory of Ranked Signals (STARS) has been used to target driver AF drivers with ablation and produced some promising initial results [47]. GC-based analysis of AF mapping data has been utilised to identify dominant excitation patterns [48], however, ours is the only study that utilises it to quantify organisation of fibrillation and localise RDs, and was validated against high-resolution phase analysis. Other techniques exist, specifically divergence and curl mapping, for quantitatively identifying focal and RDs. Although, the methodology was developed for analysing conduction velocity vectors from activation mapping, it could also be applied to GC vector maps for analysing fibrillation data [49].

American Heart Association.
Circulation
Arrhythmia and Electrophysiology

This study has a few important limitations. GC-vector mapping was used to analyse two-dimensional fibrillatory data and may not reflect the transmural propagation in fibrillation. EGMs show sharp deflection in fibrillation, rather than a sinusoidal waveforms seen from optical fluorescence. GC-based analysis is more dependent on determining causal relationships over time than timing of local activation, further work is needed to evaluate whether GC vector maps from EGMs and optical fluorescence are comparable. GC based analysis is used to determine both global fibrillatory organisation with CPI and to localise RDs with GC vector maps, thus, these methods are not mutually exclusive and may influence each other. One of the limitations of human VF mapping in this study was that the analysis was not performed in a whole intact ex vivo perfused heart. Nevertheless, the volume of an LV wedge preparation was clearly sufficient to sustain VF, and the volume of myocardium in the wedge preparation greater that the wavelength volume of the fibrillation [21] and therefore a suitable preparation to study fibrillatory dynamics and to validate of the GC-based analysis tools in large hearts.

Conclusion

In summary, we present novel methodologies based on GC analysis for measuring global fibrillatory organisation and mapping RDs. The techniques presented here are optimised for sequential mapping with limited spatial resolution and coverage, and were developed and validated against high-resolution phase processed optical mapping data. They were further tested in human VF and then adapted for use with intracardiac EGMs. GC-based fibrillation analysis holds potential for identifying patients with globally organised fibrillation, mapping fibrillation mechanisms and for guiding ablation therapy within the spatiotemporal constraints of current clinical mapping technology.

American Heart Association. Circulation Arrhythmia and Electrophysiology

Sources of Funding: This work was supported by the British Heart Foundation (Grants Nos. RG/16/3/32175 and PG/16/17/32069). FSN was also supported by the National Institute for Health Research (NIHR) Imperial Biomedical Research Centre, and an NIHR Clinical Lectureship (CL-2011-21-001). KA and IR acknowledge the support of the Leducq Foundation (project RHYTHM).

Disclosures: Dr B.S. Handa, Dr X Li, Prof N.S. Peters and Dr. F.S. Ng are applicants on a patent to Granger Causality Fibrillation Mapping (UK Patent Application No. 1903259.8)

References:

1. Kay MW, Walcott GP, Gladden JD, Melnick SB, Rogers JM. Lifetimes of Epicardial Rotors in Panoramic Optical Maps of Fibrillating Swine Ventricles. *Am J Physiol Heart Circ Physiol.* 2006;291:1935–1941.

2. Gutbrod SR, Walton R, Gilbert S, Meillet V, Jaïs P, Hocini M, Haïssaguerre M, Dubois R, Bernus O, Efimov IR . Quantification of the Transmural Dynamics of Atrial Fibrillation by Simultaneous Endocardial and Epicardial Optical Mapping in an Acute Sheep Model. *Circ Arrhythm Electrophysiol*. 2015;8:456–465.

3. Csepe TA, Hansen BJ, Fedorov VV. Atrial fibrillation driver mechanisms: Insight from the isolated human heart. *Trends Cardiovasc Med*. 2017;27:1–11.

4. Hansen BJ, Zhao J, Li N, Zolotarev A, Zakharkin S, Wang Y, Atwal J, Kalyanasundaram A, Abudulwahed SH, Helfrich KM, et al. Human Atrial Fibrillation Drivers Resolved With Integrated Functional and Structural Imaging to Benefit Clinical Mapping. *JACC Clin Electrophysiol.* 2018;4:1501–1515.

5. Calkins H, Hindricks G, Cappato R, Kim YH, Saad EB, Aguinaga L, Akar JG, Badhwar V, Brugada J, Camm J, et al. 2017 HRS/EHRA/ECAS/APHRS/SOLAECE expert consensus statement on catheter and surgical ablation of atrial fibrillation: Executive summary. *Europace.* 2018;20:157–208.

6. Narayan SM, Baykaner T, Clopton P, Schricker A, Lalani GG, Krummen DE, Shivkumar K, Miller JM. Ablation of rotor and focal sources reduces late recurrence of atrial fibrillation compared with trigger ablation alone: extended follow-up of the CONFIRM trial (Conventional Ablation for Atrial Fibrillation With or Without Focal Impulse and Rotor Modulat. *J Am Coll Cardiol*. 2014;63:1761–1768.

7. Haïssaguerre M, Hocini M, Cheniti G, Duchateau J, Sacher F, Puyo S, Cochet H, Takigawa M, Denis A, Martin R, et al. Localized Structural Alterations Underlying a Subset of Unexplained Sudden Cardiac Death. *Circ Arrhythm Electrophysiol.* 2018;11:e006120.

American Heart Association.
Circulation
Arrhythmia and Electrophysiology

8. De Groot N, van der Does L, Yaksh A, Lanters E, Teuwen C, Knops P, van de Woestijne P, Bekkers J, Kik C, Bogers A, et al. Direct Proof of Endo-Epicardial Asynchrony of the Atrial Wall During Atrial Fibrillation in Humans. *Circ Arrhythm Electrophysiol*. 2016;9:1–7.

9. Ho G, Villongco CT, Yousefian O, Bradshaw A, Nguyen A, Faiwiszewski Y, Hayase J, Rappel WJ, McCulloch AD, Krummen DE. Rotors exhibit greater surface ECG variation during ventricular fibrillation than focal sources due to wavebreak, secondary rotors, and meander. *J Cardiovasc Electrophysiol*. 2017;28:1158–1166.

10. Cantwell CD, Roney CH, Ng FS, Siggers JH, Sherwin SJ, Peters NS. Techniques for automated local activation time annotation and conduction velocity estimation in cardiac mapping. *Comput Biol Med*. 2015;65:229–242.

11. Nattel S, Xiong F, Aguilar M. Demystifying rotors and their place in clinical translation of atrial fibrillation mechanisms. *Nat Rev Cardiol.* 2017;14:509-520.

12. Umapathy K, Nair K, Masse S, Krishnan S, Rogers J, Nash MP, Nanthakumar K. Phase Mapping of Cardiac Fibrillation. *Circ Arrhythm Electrophysiol*. 2010;3:105–114.

13. Roney CH, Cantwell CD, Bayer JD, Qureshi NA, Lim PB, Tweedy JH, Kanagaratnam P, Peters NS, Vigmond EJ, Ng FS. Spatial Resolution Requirements for Accurate Identification of Drivers of Atrial Fibrillation. *Circ Arrhythm Electrophysiol*. 2017;10:e004899.

14. Balasundaram K, Umapathy K, Jeyaratnam J, Niri A, Massé S, Farid T, Nair K, Asta J, Cusimano RJ, Vigmond E, et al. Tracking Rotors With Minimal Electrodes. *Circ Arrhythm Electrophysiol*. 2015;8:447–455.

15. Martinez-Mateu L, Romero L, Ferrer-Albero A, Sebastian R, Rodríguez Matas JF, Jalife J, Berenfeld O, Saiz J. Factors affecting basket catheter detection of real and phantom rotors in the atria: A computational study. *PLOS Comput Biol*. 2018;14:e1006017.

16. Laughner J, Shome S, Child N, Shuros A, Neuzil P, Gill J, Wright M. Practical Considerations of Mapping Persistent Atrial Fibrillation With Whole-Chamber Basket Catheters. *JACC Clin Electrophysiol*. 2016;2:55–65.

17. Bear LR, Le Grice IJ, Sands GB, Lever NA, Loiselle DS, Paterson DJ, Cheng LK, Smaill BH. How Accurate Is Inverse Electrocardiographic Mapping? A Systematic In Vivo Evaluation. *Circ Arrhythm Electrophysiol*. 2018;11:e006108.

18. Cuculich PS, Wang Y, Lindsay BD, Faddis MN, Schuessler RB, Damiano RJ Jr, Li L, Rudy Y. Noninvasive characterization of epicardial activation in humans with diverse atrial fibrillation patterns. *Circulation.* 2010;122:1364–1372.

19. Wang Y, Cuculich PS, Zhang J, Desouza KA, Vijayakumar R, Chen J, Faddis MN, Lindsay BD, Smith TW, Rudy Y. Noninvasive electroanatomic mapping of human ventricular

American Heart Association.
Circulation
Arrhythmia and Electrophysiology

arrhythmias with electrocardiographic imaging. *Sci Transl Med*. 2011;3:98ra84.

20. Granger CWJ. Investigating Causal Relations by Econometric Models and Cross-spectral Methods. *Econometrica*. 1969;37:424–438.

21. Aras KK, Faye NR, Cathey B, Efimov IR. Critical Volume of Human Myocardium Necessary to Maintain Ventricular Fibrillation. *Circ Arrhythm Electrophysiol.* 2018;11:e006692.

22. Ng FS, Kalindjian JM, Cooper SA, Chowdhury RA, Patel PM, Dupont E, Lyon AR, Peters NS. Enhancement of Gap Junction Function During Acute Myocardial Infarction Modifies Healing and Reduces Late Ventricular Arrhythmia Susceptibility. *JACC Clin Electrophysiol.* 2016;2:574–582.

23. Ng FS, Shadi IT, Peters NS, Lyon AR. Selective heart rate reduction with ivabradine slows ischaemia-induced electrophysiological changes and reduces ischaemia–reperfusion-induced ventricular arrhythmias. *J Mol Cell Cardiol*. 2013;59:67–75.

24. Granger CWJ. Investigating Causal Relations by Econometric Models and Cross-spectral Methods. *Econometrica*. 1969;37:424–438

25. Silvapulle P, Choi J-S. Testing for linear and nonlinear granger causality in the stock price-volume relation: Korean evidence. *Q Rev Econ Financ*. 1999;39:59–76.

26. Handa BS, Roney CH, Houston C, Qureshi NA, Li X, Pitcher DS, Chowdhury RA, Lim PB, Dupont E, Niederer SA, et al. Analytical approaches for myocardial fibrillation signals. *Comput Biol Med*. 2018;102:315–326

27. Roney CH, Cantwell CD, Qureshi NA, Chowdhury RA, Dupont E, Lim PB, Vigmond EJ, Tweedy JH, Ng FS, Peters NS. Rotor Tracking Using Phase of Electrograms Recorded During Atrial Fibrillation. *Ann Biomed Eng*. 2017;45:910–923.

28. Ganesan AN, Kuklik P, Lau DH, Brooks AG, Baumert M, Lim WW, Thanigaimani S, Nayyar S, Mahajan R, Kalman JM, et al. Bipolar electrogram Shannon entropy at sites of rotational activation implications for ablation of atrial fibrillation. *Circ Arrhythm Electrophysiol.* 2013;6:48–57.

29. Haïssaguerre M, Jaïs P, Shah DC, Takahashi A, Hocini M, Quiniou G, Garrigue S, Le Mouroux A, Le Métayer P, Clémenty J. Spontaneous Initiation of Atrial Fibrillation by Ectopic Beats Originating in the Pulmonary Veins. *N Engl J Med.* 1998;339:659–666.

30. Haissaguerre M, Vigmond E, Stuyvers B, Hocini M, Bernus O. Ventricular arrhythmias and the His-Purkinje system. *Nat Rev Cardiol*. 2016;13:155–166.

31. Moe GK, Rheinboldt WC, Abildskov JA. A computer model of atrial fibrillation. *Am Heart J.* 1964;67:200–220.

American Heart Association. Circulation Arrhythmia and Electrophysiology

32. Chen J, Mandapati R, Berenfeld O, Skanes AC, Gray RA, Jalife J. Dynamics of wavelets and their role in atrial fibrillation in the isolated sheep heart. *Cardiovasc Res*. 2000;48:220–232.

33. Reumann M, Bohnert J, Osswald B, Hagl S, Doessel O. Multiple wavelets, rotors, and snakes in atrial fibrillation—a computer simulation study. *J Electrocardiol*. 2007;40:328–334.

34. Allessie MA, de Groot NM, Houben RP, Schotten U, Boersma E, Smeets JL, Crijns HJ. Electropathological substrate of long-standing persistent atrial fibrillation in patients with structural heart disease longitudinal dissociation. *Circ Arrhythm Electrophysiol*. 2010;3:606–615.

35. Verheule S, Eckstein J, Linz D, Maesen B, Bidar E, Gharaviri A, Schotten U. Role of endo-epicardial dissociation of electrical activity and transmural conduction in the development of persistent atrial fibrillation. *Prog Biophys Mol Biol*. 2014;115:173–185.

36. Haissaguerre M, Hocini M, Shah AJ, Derval N, Sacher F, Jais P, Dubois R. Noninvasive panoramic mapping of human atrial fibrillation mechanisms: A feasibility report. *J Cardiovasc Electrophysiol*. 2013;24:711–717.

37. Narayan SM, Krummen DE, Shivkumar K, Clopton P, Rappel W-J, Miller JM. Treatment of Atrial Fibrillation by the Ablation of Localized Sources. *J Am Coll Cardiol*. 2012;60:628–636.

38. King B, Porta-Sánchez A, Massé S, Zamiri N, Balasundaram K, Kusha M, Jackson N, Haldar S, Umapathy K, Nanthakumar K. Effect of spatial resolution and filtering on mapping cardiac fibrillation. *Heart Rhythm.* 2017;14:608–615.

39. Vijayakumar R, Vasireddi SK, Cuculich PS, Faddis MN, Rudy Y. Methodology Considerations in Phase Mapping of Human Cardiac Arrhythmias. *Circ Arrhythm Electrophysiol*. 2016;9:e004409.

40. Seth AK, Barrett AB, Barnett L. Granger Causality Analysis in Neuroscience and Neuroimaging. *J Neurosci*. 2015;35:3293–3297.

41. Brovelli A, Ding M, Ledberg A, Chen Y, Nakamura R, Bressler SL. Beta oscillations in a large-scale sensorimotor cortical network: Directional influences revealed by Granger causality. *Proc Natl Acad Sci USA*. 2004;101:9849–9854.

42. Papagiannopoulou C, Decubber S, Miralles DG, Demuzere M, Verhoest NEC, Waegeman W. Analyzing Granger causality in climate data with time series classification methods. *Jt Eur Conf Mach Learn Knowl Discov Databases*. 2017;1–12.

43. Nash MP, Mourad A, Clayton RH, Sutton PM, Bradley CP, Hayward M, Paterson DJ, Taggart P. Evidence for Multiple Mechanisms in Human Ventricular Fibrillation. *Circulation*. 2006;114:536–542.

44. Lee S, Sahadevan J, Khrestian CM, Cakulev I, Markowitz A, Waldo AL. Simultaneous

American Heart Association
Circulation
Arrhythmia and Electrophysiology

Biatrial High-Density (510-512 Electrodes) Epicardial Mapping of Persistent and Long-Standing Persistent Atrial Fibrillation in Patients: New Insights Into the Mechanism of Its Maintenance. *Circulation*.132:2108–2117.

45. Lee G, Kumar S, Teh A, Madry A, Spence S, Larobina M, Goldblatt J, Brown R, Atkinson V, Moten S, et al. Epicardial wave mapping in human long-lasting persistent atrial fibrillation: transient rotational circuits, complex wavefronts, and disorganized activity. *Eur Heart J*. 2014;35:86–97.

46. Tao S, Way SF, Garland J, Chrispin J, Ciuffo LA, Balouch MA, Nazarian S, Spragg DD, Marine JE, Berger RD, et al. Ablation as targeted perturbation to rewire communication network of persistent atrial fibrillation. *PLoS One*. 2017;12:1–18.

47. Honarbakhsh S, Hunter RJ, Ullah W, Keating E, Finlay M, Schilling RJ. Ablation In Persistent Atrial Fibrillation Using The Stochastic Trajectory Analysis Of Ranked Signals (STAR) Mapping Method. *JACC Clin Electrophysiol.* 2019;5:817-829.

48. Rodrigo M, Climent AM, Liberos A, Calvo D, Fernández-Avilés F, Berenfeld O, Atienza F, Guillem MS. Identification of Dominant Excitation Patterns and Sources of Atrial Fibrillation by Causality Analysis. *Ann Biomed Eng.* 2016;44:2364–2376.

49. Dallet C, Roney C, Martin R, Kitamura T, Puyo S, Duchateau J, Dumas-Pomier C, Ravon G, Bear L, Derval N, et al. Cardiac propagation pattern mapping with vector field for helping tachyarrhythmias diagnosis with clinical tridimensional electro-anatomical mapping tools. *IEEE Trans Biomed Eng.* 2019;66:373–382.

American Heart Association.
Circulation
Arrhythmia and Electrophysiology

Figure Legends:

Figure 1. Novel tools for measuring organisation and localising areas with RDs. **(A)** An example simplified 3 x 3 pixel grid with corresponding optical mapping signals below showing GC vectors between signals with the strongest temporal dependence. **(B)** Causality pairing index for data in (A) showing 4 pixel pairs with causal temporal dependence of propagation (shaded black, corresponding to the GC vectors). CPI was defined as the pixel pairs with causal dependence divided by all possible pixel pairings in a data set. **(C)** An example dominant frequency (DF)

map showing all the DFs driving fibrillation in the LV anterior wall. The histogram below plots these frequencies. The frequency dominance index (FDI) is defined as the proportion of area in fibrillation occupied by the highest amplitude DF in the global spectrum divided by the relative area of all frequencies in the mapped area (black arrow). **(D)** Circulatory interdependence value (CIV) for 3 examples. For each GC vector (dark blue), a cross product virtual 3D vector was generated (green arrow) relative to vector to the centre (dashed black line). The resulting virtual 3D vector was binarised as pointing up or down as shown in example for vector a and vector b. CIV was calculated on a scale of 0 to 1 by subtracting the number of vectors pointing down from no of vectors pointing up divided by total number of vectors. The three example above demonstrate expected values for; example 1- stable RD, example 2 – random propagation, example 3 – linearly propagating wavefront. With this method areas of rotational activity will give a CIV value closer to 1.

American Heart Association.
Circulation
Arrhythmia and Electrophysiology

Figure 2. The causality pairing index (CPI) and frequency dominance index (FDI) can characterise the global organisation of fibrillation at low spatial resolution. Graphs showing negative correlation between a measure of disorganisation and instability, the number of locations occupied by non-significant short lived PSs (l_{ps}) and CPI **(A)** and l_{ps} and FDI **(B),** and no correlation between l_{ps} and Shannon entropy (Sh_{en}) **(C),** at decreasing resolutions of 50% (left), 25% (middle) and 12.5% (right) of full spatial resolution from optical mapping of rat VF. Non-significant PSs were defined as PSs with <2 rotations and RD were defined as >2 rotations. (*Linear regression analysis, F-Test, coefficients of determination - R^2 and p values are indicated, n =18*)

Figure 3. Quantifying global organisation in fibrillation infers the likely underlying mechanism. Representative data sets of rat VF selected from organisational analysis categorisation of fibrillation as low (left), intermediate (middle) and organised (right) from Figure 2. **(A)** RD heat map showing incidence of significant RDs (≥ 2 rotations) **(B)** The respective global dominant frequency histogram with frequency dominance index (FDI) value and **(C)** graphs showing characterisation of RD for each data set [l_{ps} – number of locations/pixels occupied by non-significant PS with <2 rotation, l_r - no. of location occupied by significant RDs with ≥ 2 rotations, max (n_r) - maximum rotations for a single significant RDs]

Figure 4. Granger causality (GC) mapping can be utilised for analysing fibrillation data. An example GC vector map of an organised rat VF heart showing neighbouring regions with causal interdependence **(A)**, and zoomed localisation of a driver region showing a signature continuous circular interdependence of neighbouring GC vectors **(B)**, with correlating optical mapping signals from the driver region showing repetitive sequential activation **(C).** *(Data analysed at 25% of full spatial resolution, correlating RD heat map in Figure 5 – Heart C)*

American Heart Association
Circulation
Arrhythmia and Electrophysiology

Figure 5. GC vector mapping can reliably localise and differentiate between areas harbouring RDs and areas without RDs in VF. **(A)** RD heat maps constructed from three organised rat VF data sets; Heart A, B and C (left) and the correlating limited coverage GC vector maps showing driver regions with continuous circular interdependence of GC vectors with one directional flow of GC-vectors (blue) and non-driver regions showing no circular interdependence of GC vectors. Correlating CIV values between 0 (minimum) and maximum (1) for each respective region listed below. (Data analysed at 25% of full spatial resolution). **(B)** A

graph showing CIV value of driver regions versus non-driver regions with sample GC maps (left, heart C). (*T-test, n=3, p=0.0002*)

Figure 6. GC based analysis of human VF quantifies global fibrillatory organisation and maps underlying mechanism. **(A)** Graphs showing negative correlation between l_{ps} and FDI at decreasing spatial resolution. **(B)** Full spatial resolution RD heat maps in VF of LV wedge epicardial recordings with corresponding GC vector maps at 25% spatial resolution from two representative hearts above (Heart A and Heart B)**.** **(***Linear regression analysis, F-Test, coefficients of determination - R^2 and p values are indicated, data from 33 VF recordings, n =12)*

American Heart Association.
Circulation
Arrhythmia and Electrophysiology

Figure 7. GC vector maps generated from intracardiac EGMs acquired with multipolar catheters. **(A)** A-FocusII^{TM} mapping catheter three-dimensional electrode (red dot) spatial configuration with corresponding bipoles (blue triangle) within the atrium (left). EGMs processing for GC analysis (right) - (i) Sample raw bipolar EGM (ii) 40-250Hz band-pass filtering and low-pass filtering of signals <25Hz (iii) signal rectification (iv) down-sampling. **(B)** Representative GC vector map for a paced rhythm mapped by the catheter (left) and correlating raw and rectified EGMs (right).

Figure 8. GC vector maps can localise RDs from intracardiac EGMs acquired with a multipolar catheter. Representative GC vector maps for a RD positive site with high CIV value (top) and RD negative site with low CIV value (bottom), corresponding **(B)** CIV values over time and **(C)** EGMs. *dashed line = cut off for a RD-positive site. **(D)** AF mapping data from 16 persistent AF

patients showing the number of kernels and windows with RDs. **(E)** Graph showing the positive correlation between CPI and number of RDs. (*Linear regression analysis, F-Test, coefficients of determination -* R^2 *and p value is indicated, n =16*)

American Heart Association.
Circulation
Arrhythmia and Electrophysiology

What is Known?

- Pre-clinical studies have implicated multiple competing mechanisms for sustaining myocardial fibrillation.
- Clinical translation to guide treatment in patients with AF and VF survivors remains challenging due to the poor spatial resolution of clinical mapping systems and a lack of suitable analysis tools.

What this Study Adds?

- Granger causality (GC) analysis, originally an econometric tool for quantifying causal relationships between complex time-series, was developed in rat VF, and validated in human VF and AF as a novel fibrillation mapping tool.
- GC-based fibrillation analysis can measure global fibrillation organisation, characterise dominant propagating patterns and map rotational drivers using low spatial resolution sequentially-acquired data.

American Heart Association.
Circulation
Arrhythmia and Electrophysiology

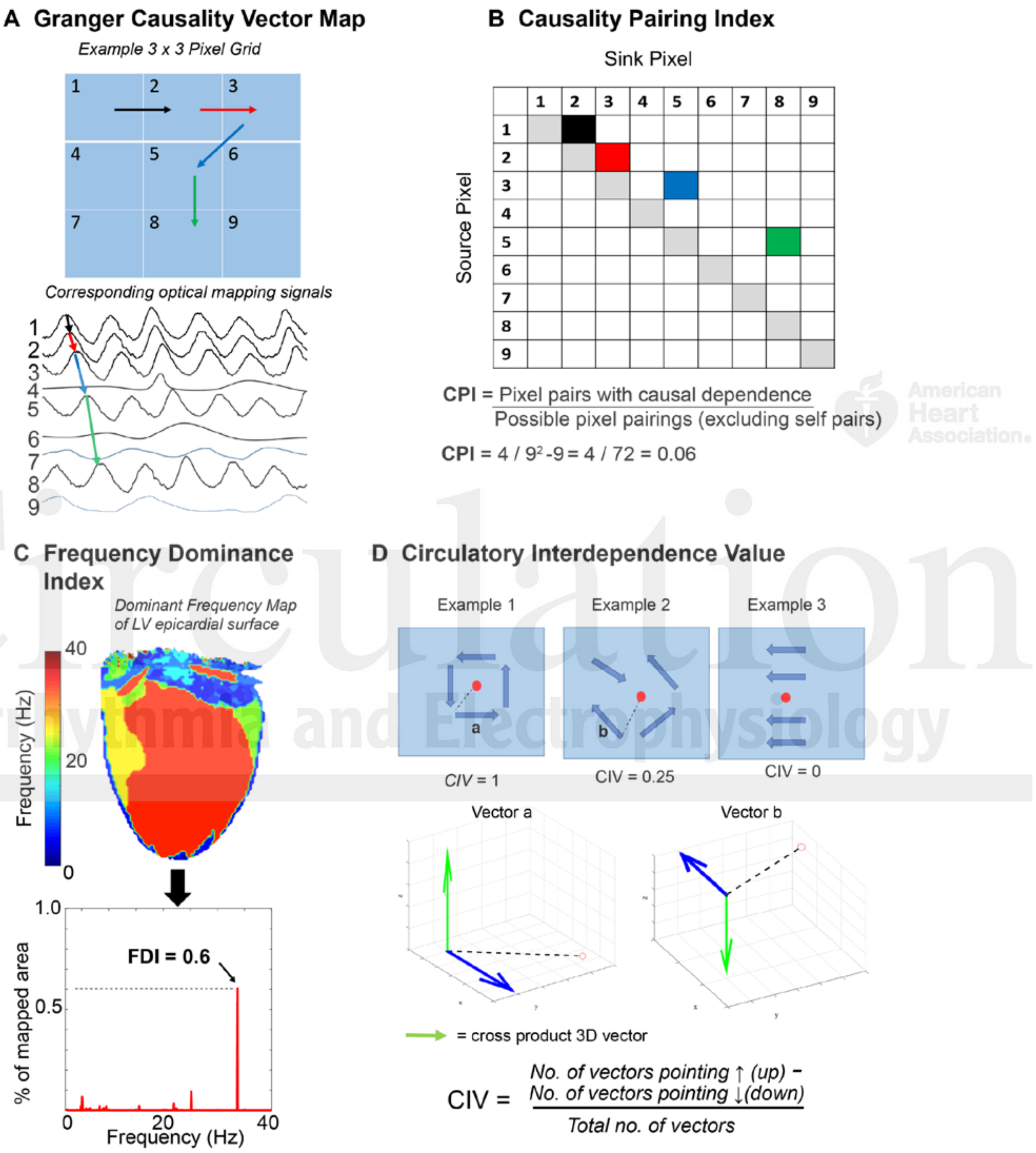

American Heart Association.
Circulation
Arrhythmia and Electrophysiology

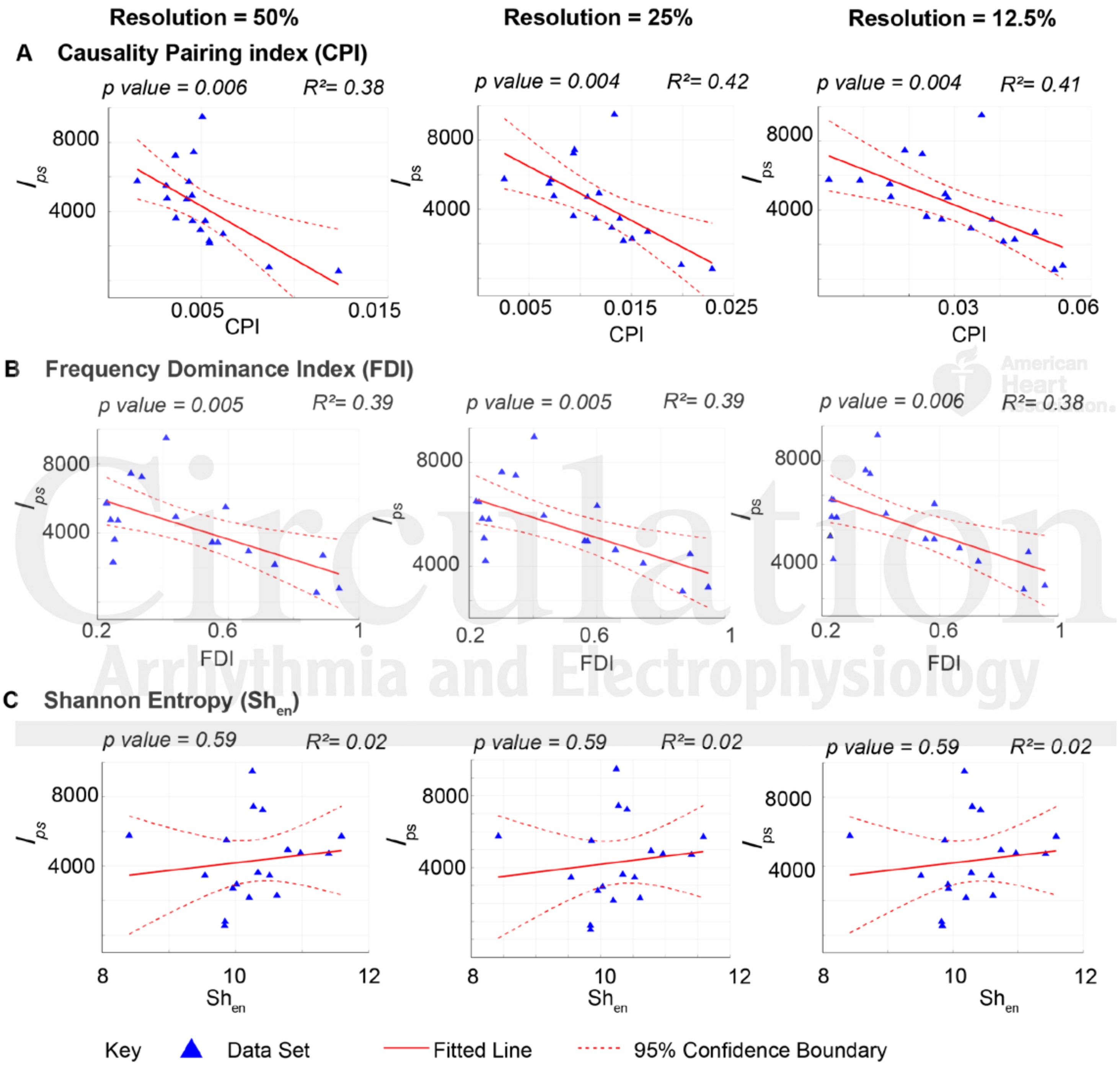

Circulation Arrhythmia and Electrophysiology
American Heart Association

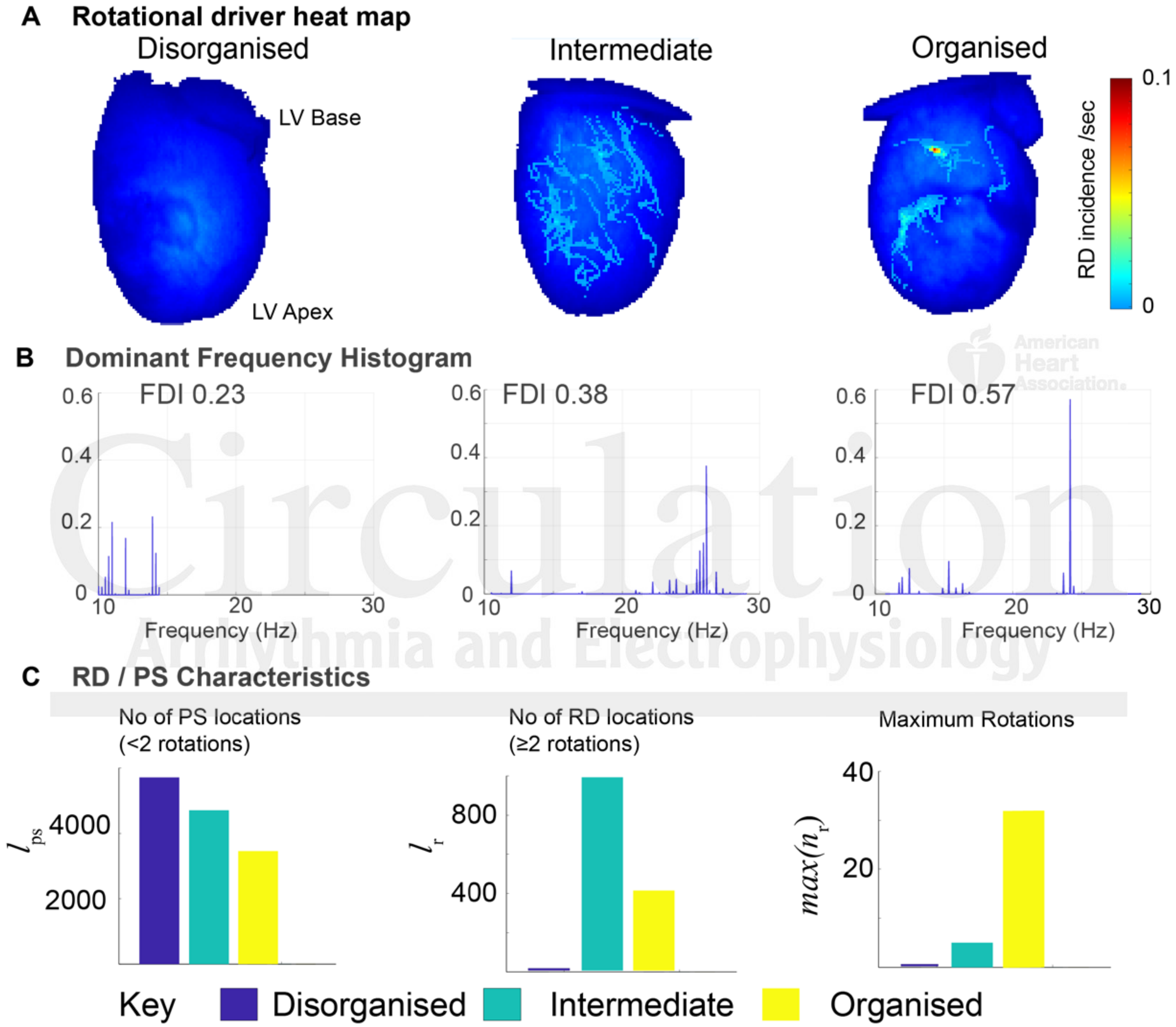

American Heart Association
Circulation
Arrhythmia and Electrophysiology

Granger causality vector mapping in VF

Resolution = 25%

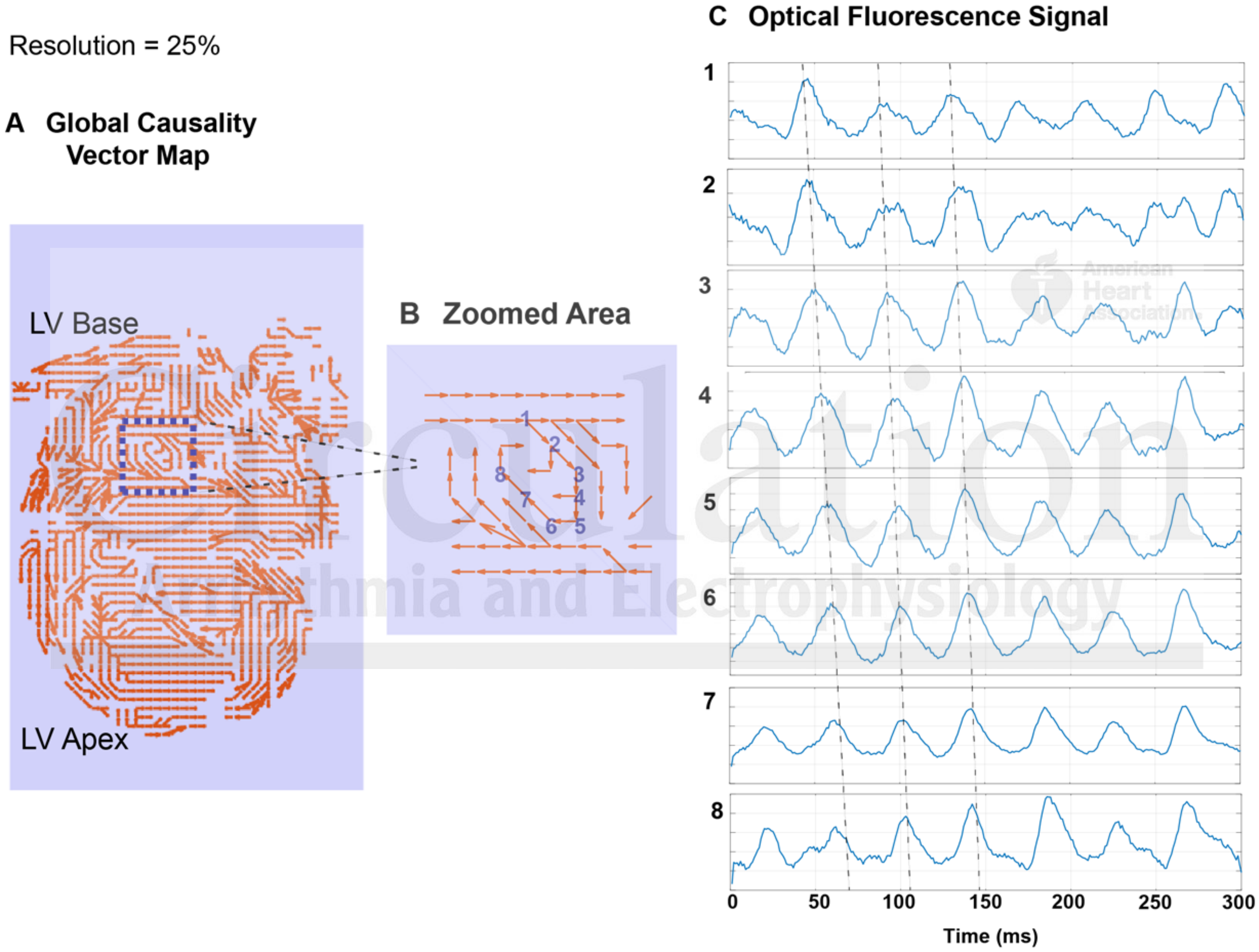

Circulation Arrhythmia and Electrophysiology
American Heart Association

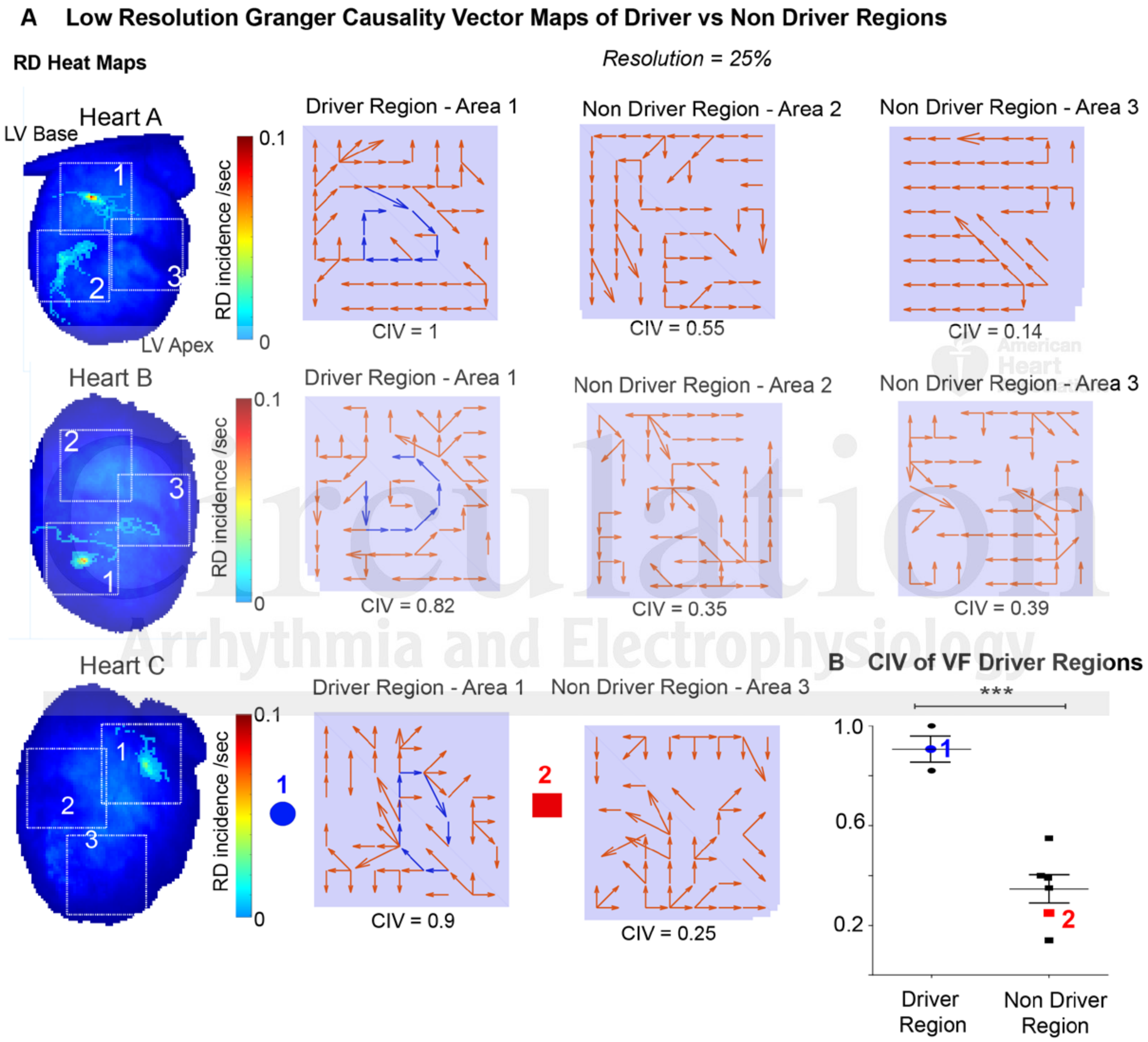

Circulation Arrhythmia and Electrophysiology

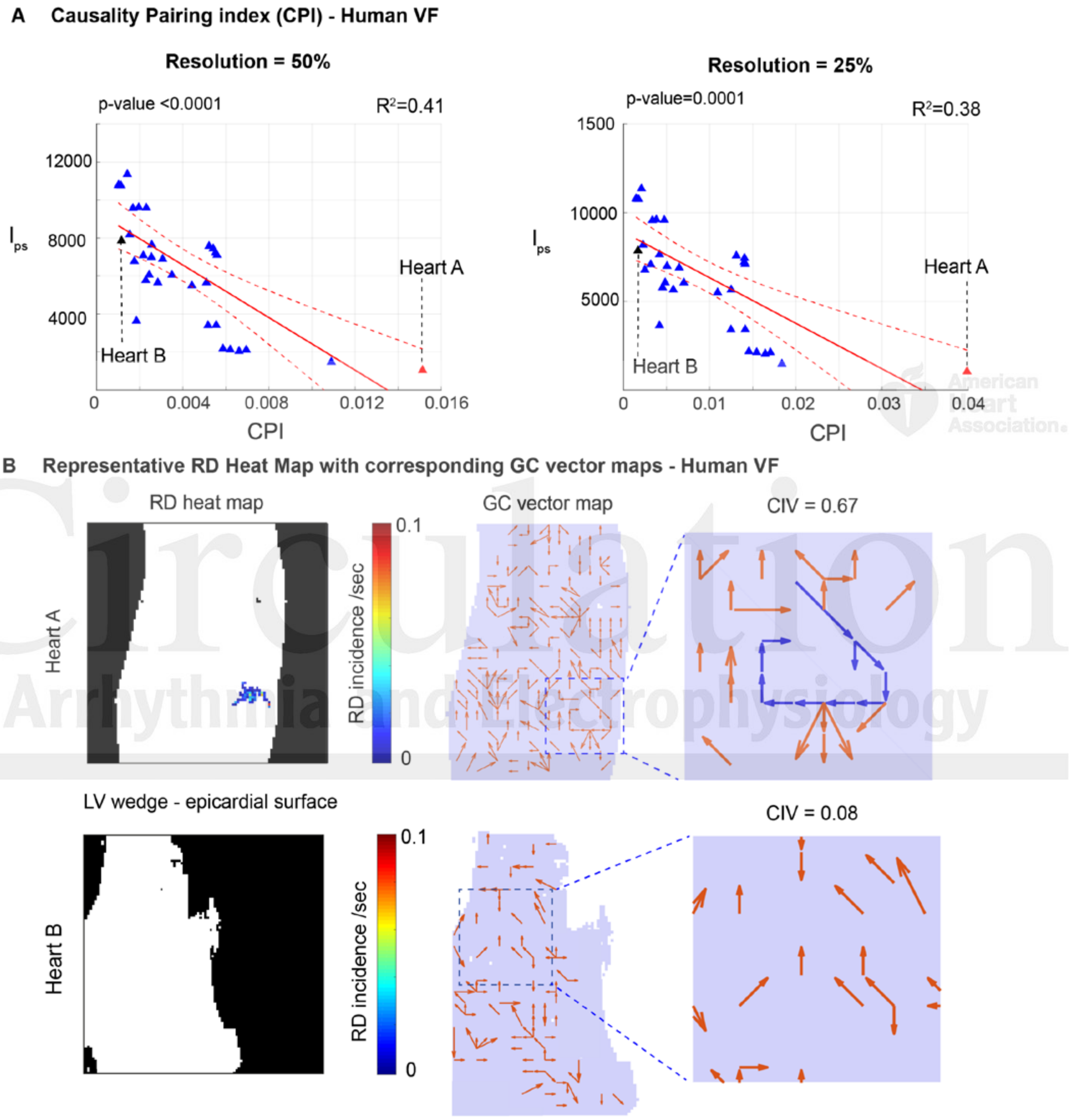

Circulation Arrhythmia and Electrophysiology
American Heart Association

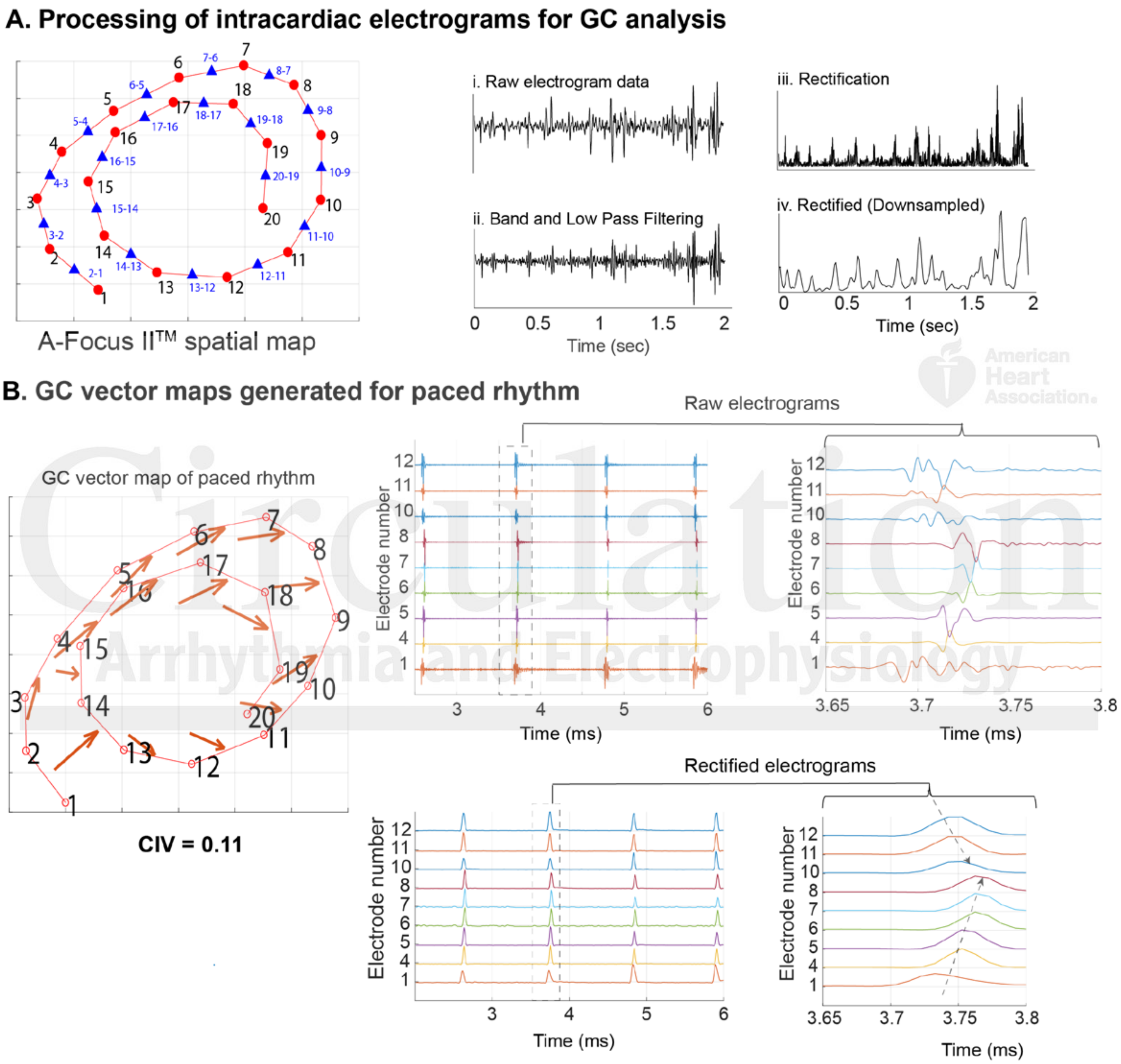

American Heart Association
Circulation Arrhythmia and Electrophysiology

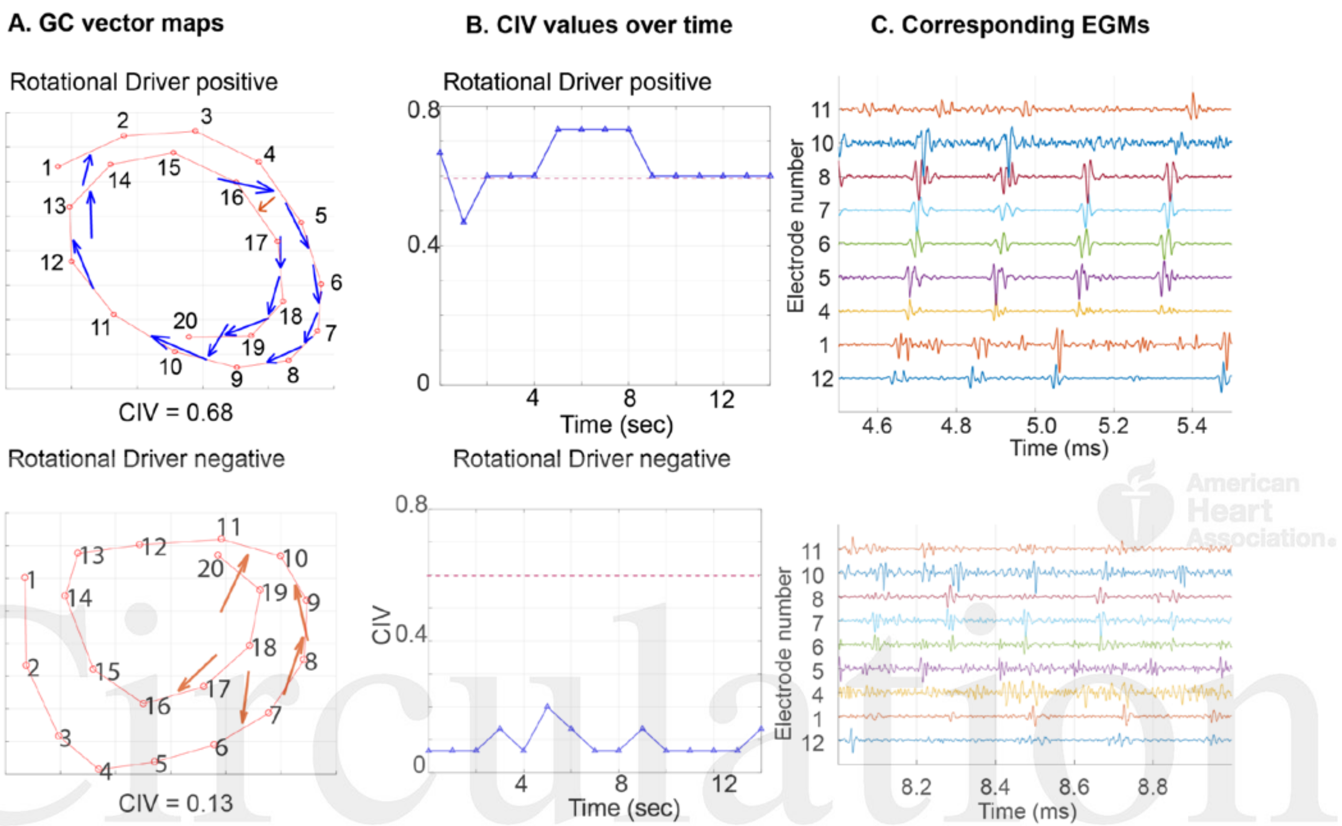

D. Summary data of PsAF patients

Patient ID	Total Kernels	Total Windows	RD +ve windows	RD+Ve kernel
1	27	616	1	1
2	25	364	10	4
3	21	649	0	0
4	20	532	0	0
5	19	420	0	0
6	16	346	1	1
7	15	402	2	1
8	15	316	2	1
9	13	213	0	0
10	12	341	2	2
11	10	329	0	0
12	9	281	5	2
13	9	193	0	0
14	9	222	0	0
15	8	171	0	0
16	8	200	5	2
**Total**	**236**	**5595**	**28**	**14**
**Average**	**14.8**	**349.7**	**1.8**	**0.9**
**Percentage of total**			0.50	5.93

*RD - Rotational Driver, +ve = positive,
*Kernel - a single locational AFocus EGM recording
*Window = 8-sec overlapping electrogram time windows with a window-shift of 1 sec

E. CPI and number of RD kernels

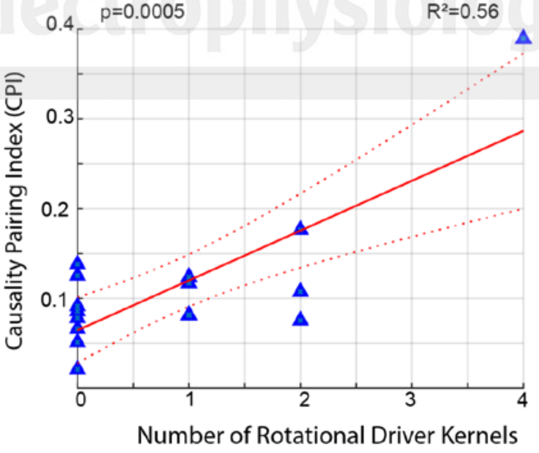

Circulation Arrhythmia and Electrophysiology
American Heart Association

VISUAL ABSTRACT

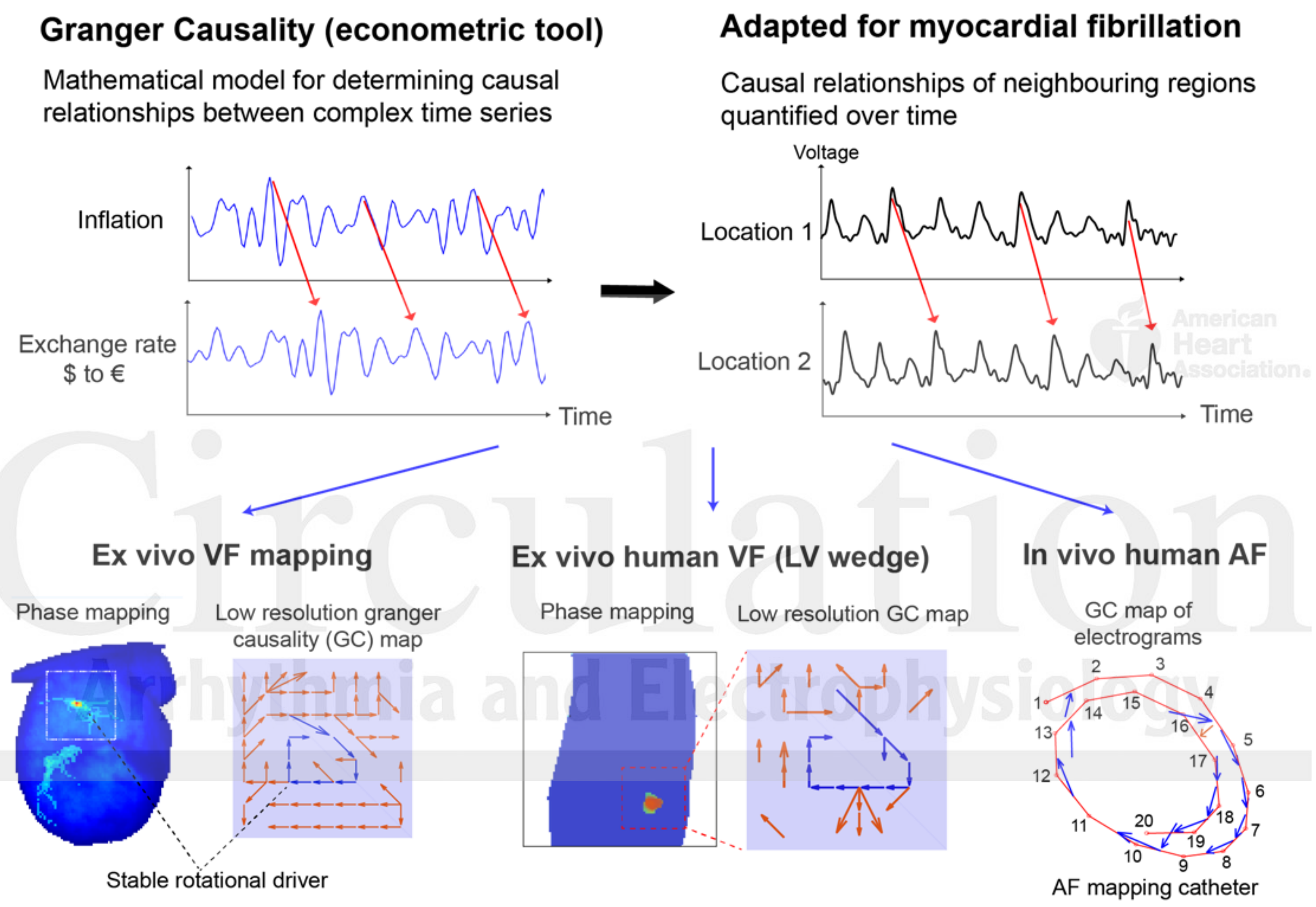

Circulation Arrhythmia and Electrophysiology
American Heart Association

Key Features:

(1) Measure global fibrillatory organisation using data with low spatial resolution
(2) Localise drivers in cases of organised AF/ VF.
