# Supplementary figures and images for: Granger Causality–Based Analysis for Classification of Fibrillation Mechanisms and Localization of Rotational Drivers
Source: Circ Arrhythm Electrophysiol. 2020 Feb 16;13(3):e008237. doi: 10.1161/CIRCEP.119.008237 (PMC7069398; doi:10.1161/CIRCEP.119.008237)

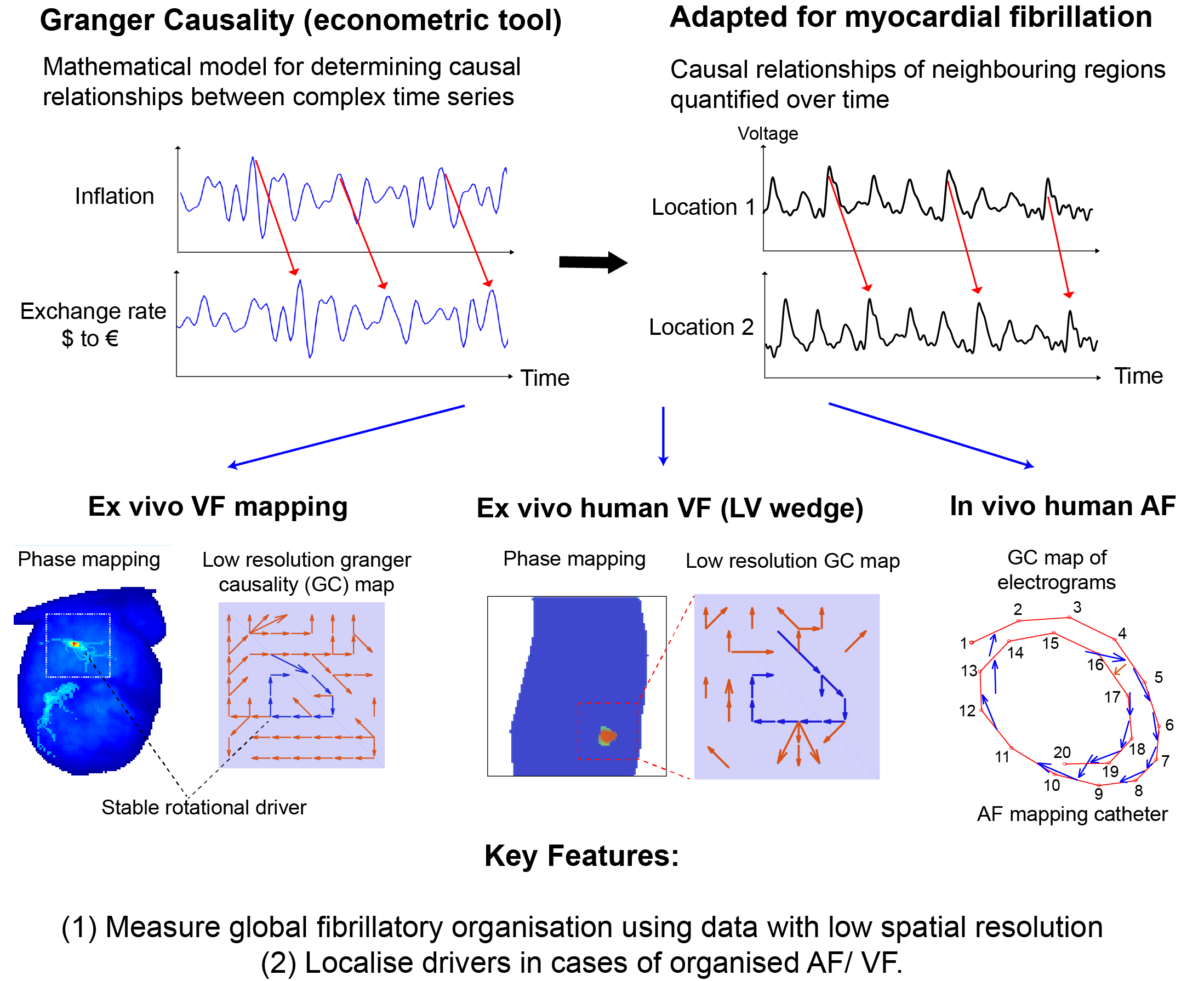

Supplement: Supplementary file 3 [file hae-13-e008237-s003.tif]
